# Evolutionary games, climate and the generation of diversity

**DOI:** 10.1371/journal.pone.0184052

**Published:** 2017-08-31

**Authors:** Daniel Friedman, Jacopo Magnani, Dhanashree Paranjpe, Barry Sinervo

**Affiliations:** 1 Economics Department, University of California Santa Cruz, Santa Cruz, CA, United States of America; 2 Division of Social Science, New York University Abu Dhabi, Abu Dhabi, United Arab Emirates; 3 Ecology and Evolutionary Biology Dept, University of California Santa Cruz, Santa Cruz, CA, United States of America; University of California Santa Barbara Counseling Services, UNITED STATES

## Abstract

Environmental stochasticity and climate affect outcomes in evolutionary games, which can thereby affect biological diversity. Our maximum likelihood (ML) estimates of replicator dynamics for morph frequency data from control (25 years) and three experimentally perturbed populations (14 years) of side-blotched lizards yield a 3 × 3 payoff matrix in the generalized Rock-Paper-Scissors family; it has intransitive best replies, and each strategy is its own worst reply. ML estimates indicate significant interactive effects of density and temperature on morph frequency. Implied dynamics feature a powerful interior attractor and recover (for the first time) observed 4-5 year oscillations. Our evolutionary experiment on morph frequency confirms that oscillations are driven by frequency dependent selection, but climate entrains the cycles across the perturbed and control populations within 10 generations. Applying the model across the species range, we find that climate also accounts for morph fixation and mating system diversity, suggesting climate may similarly impact ecosystem diversity.

## Introduction

Frequency dependent selection occurs when biological fitness depends on relative prevalence of alternative strategies. A famous example occurs among throat-color morphs of side-blotched lizards, where despotic orange defeats cooperative blue, which defeats sneaker yellow, which defeats orange, in rock-paper-scissors (RPS) cycles [1]. Game theory has been applied to understand the role of frequency dependent selection in diverse biological systems [2] [3], [4]. The basic idea is to summarize the fitness impact of morph *j* on morph *i* in a *n* × *n* payoff matrix *W* = ((*W*_*ij*_))_*i*,*j* = 1,…,*n*_. Previous work with the side-blotched lizard, *Uta stansburiana* focused on siring success using bivariate analysis.

Do we indeed get RPS cycles when we take a broader view of fitness? In this paper we exploit a novel experimentally perturbed data set and new statistical techniques to re-examine payoff matrices and morph dynamics among side-blotched lizards. The new results confirm the basic cyclic domination pattern of RPS, but the dynamics provide new insight into the relatively short observed cycles. The estimation results have implications for the impact of climate on diversity at both short and long spatial scales.

**Fig 1:**
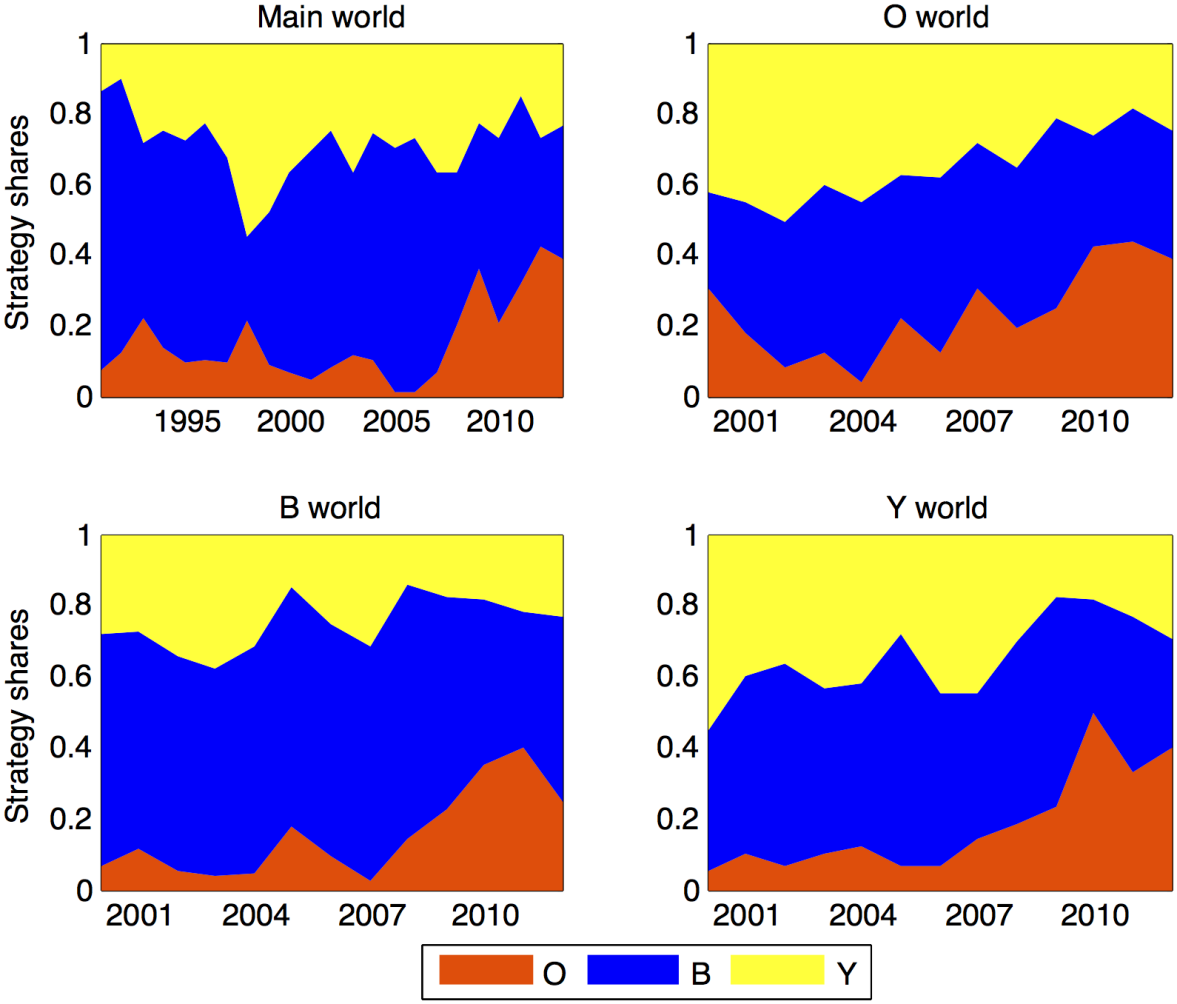
OBY allele panel. Fractions of observed OBY alleles observed among males in four *Uta stansburiana* populations.

The data set includes a natural population of side-blotched lizards called Main World (MW) for which we have a continuous record of morph frequency from 1990-2013. It also includes three experimental populations. In 1999 we seeded three rock outcrops with progeny from controlled laboratory crosses, and designated them as Orange World (OW), Blue World (BW) and Yellow World (YW) based on the color allele that was made most common [5]. Experimentally altering morph frequencies to different frequencies in a single year allows us to identify impacts of exogenous factors like weather on the temporal / spatial autocorrelation among populations. Data are plotted in Fig 1, and are available as S1 Data.

Empirical work to date has relied mainly on bivariate analysis, e.g., the siring success of each male morph is regressed on the frequency of the alternative morphs in the immediate neighborhood, and the estimated coefficients are reported as the rows of the payoff matrix [1], [6]. Here we present new methods for estimating payoff matrices, using the time series of allele frequencies in the overall population and a broader definition of fitness that subsumes all factors that may change the male morph frequencies from one generation to the next. We obtain maximum likelihood estimates for a structural model in which changes in allele frequencies are governed by standard discrete time replicator dynamics, together with stochastic disturbances following a Dirichlet distribution. Natural extensions of the structural model allow us to estimate the impact of exogenous covariates such as heat spells [7] that might limit progeny recruitment in a morph specific fashion, and endogenous covariates such as a female population density game that is linked to the male RPS game [8]. In S1 Text we present further extensions, e.g., to logistic and other error structures, and to biparental models of trait transmission. The new payoff matrix estimates differ qualitatively from earlier estimates using less inclusive techniques, and imply that climate synchronizes populations at small spatial scales (reducing diversity), but accounts for morph fixation and mating system diversity at large scales.

Color expression on the throat and flanks of male and female side-blotched lizards appears to have a simple Mendelian pattern of inheritance in which color alleles additively contribute to phenotype [9]; supporting evidence includes controlled laboratory crosses [5], field pedigree [10], [11], linkage disequilibrium gene mapping studies [12] and theoretical models [13]. Males with orange alleles (oo, bo, yo) express an aggressive phenotype and patrol large territories with many females. The aggressive orange strategy is vulnerable to invasion by sneaker males with yellow alleles (by, yy, yo), which are female mimics and can cuckold the orange males by visiting females in their harems. The strategy of yellow is beaten by the mate guarding and cooperative strategy of blue males (bb, by) that defend adjacent territories, but aggressive orange beats blue to create a rock-paper-scissors evolutionary dynamic. Current evidence suggests that male heterozygotes express intermediate phenotypes [12] as perceived also by females in mate choice studies [9]. Here we model additive genetic effects of color alleles on phenotype payoffs, in both replicator and diploid sexual models.

## Materials and methods

### Data

Primary raw data are annual counts of individual males (and females in diploid models), by genotype and location, gathered as part of annual censuses of all adult *Uta*. Since 1990 the census has covered MW, consisting of connected rock outcroppings covering approximately 1.59 hectares near Los Banos California, and since 2000 it has also covered three separate experimental locales (OW 4.24 hectares, BW 1.20 hectares and YW 7.23 hectares). Censuses record the OBY allele expressed in each mature male and each mature female (included in the census data if they survived up to an ovarian stage when females are fertilized, follicles ≥ 5*mm*, or later). We also estimated color allele frequency for the OW (*f*_*o*_ = 0.52, *f*_*b*_ = 0.16, *f*_*y*_ = 0.32), YW (*f*_*o*_ = 0.26, *f*_*b*_ = 0.18, *f*_*y*_ = 0.56), and BW (*f*_*o*_ = 0.17, *f*_*b*_ = 0.44, *f*_*y*_ = 0.39) in 1999 from the predicted distribution of color alleles in progeny, because they were produced from controlled laboratory crosses [5]. These three worlds were named for the allele we made most prevalent in the initial progeny cohort.

Color expression begins in early March as males establish territories and peaks in early April, but the four locales are slightly out of breeding synchrony, with expression of color (and reproduction) initiated first in OW, then MW, BW, and YW. Accordingly, we counted populations in that order, ensuring that males had nearly full color expression on the first census. We also carried out second and third censuses at monthly intervals to estimate the number males missed on the first pass [10], from which we infer that the count includes approximately 98% of male recruits and 97% of female recruits, i.e., surviving males or females hatched since the previous year’s census. Full expression of female color occurs when they have oviductal eggs, 2 weeks after full male color expression (e.g., when females are receptive and ready for fertilization, follicles ≥ 5*mm*), so we held two staged captures, focussing on both sexes early and females only later as they ovulated follicles, or had near-term follicles just prior to ovulation (follicles ≥ 8*mm*). There are also a few males and females that survive for two or more seasons, but *U. stansburiana* is largely an annual at Los Banos.

The 6-element vector (*N*_*oo*_, *N*_*ob*_, …, *N*_*yy*_) of male genotype data for a given year and location can be reduced to a 3-vector of allele counts used in the replicator model for male genotype frequency simply by taking the obvious weighted sums (see Table O in S1 Text), e.g., *N*_*o*_ = 2*N*_*oo*_ + *N*_*ob*_ + *N*_*oy*_. The allele shares are then computed by dividing each component of (*N*_*o*_, *N*_*b*_, *N*_*y*_) by the sum *N* of the allele counts. Due to diploidy, the population size is *n* = *N*/2, and the density covariate for each location is defined as *n* minus its historical mean for that location. Alternatively, the set of two six-element vectors comprising genotypes of both sexes can be used in full diploid sexual model, assuming Mendelian transmission and additive effects of alleles on male phenotype payoffs. One can also compute genotypic shares (frequency) by dividing each component by the population size, which here is simply the sum *n* = *N*_*oo*_ + *N*_*bo*_ + *N*_*yo*_ + *N*_*bb*_ + *N*_*by*_ + *N*_*yy*_, for a given sex.

We used weather records collected at the California Department of Forestry Station on Gonzaga Road from 1986-2012 (station LBN: 37.053 N, 121.049 W, only 7.33 km from the main population), to estimate hourly temperature records throughout the year. However, two years (1989, 1992) were missing summer records so we interpolated data from an adjacent LSB station (Los Banos Dam site; 36.994 N, 120.93 W, and 10.77 km from the main population): we regressed the LBN station data on contemporaneous data from the LSB station and then used the fitted equation to predict the two missing data points.

For each month, we computed number of hours of restriction [7], termed *h*_*r*_, when *T*_*air*_ > *T*_*preferred*_ = 37.4 C [14]. The idea is to estimate restricted activity spells due to high air temperatures, which might impact recruitment and survival, and may affect some color morphs more than others. Their small size (≈ 0.5 g) means that hatchlings will be close to thermal equilibrium with the air temperature, particularly because they spend much of their time in shade as the temperature warms, finally retreating entirely at mid-day during the summer when temperatures exceed *T*_*preferred*_.

### Statistical procedures

We fit the data to structural models estimated via maximum likelihood (ML) techniques. The models join discrete time replicator dynamics to a stochastic structure given by the Dirichlet distribution or, alternatively, by the logistic distribution. The goal is to estimate a 3×3 fitness (or payoff) matrix *W* = ((*W*_*ij*_))_*i*,*j*=1,2,3_.

The first step is to take raw census counts (considering for the moment one locale) of adult males by morph allele *N*_*o*_, *N*_*b*_, *N*_*y*_ and to compute the relative frequencies $S_{1}=\frac{N_{o}}{N_{tot}},\;\;S_{2}=\frac{N_{b}}{N_{tot}},$ and $S_{3}=\frac{N_{y}}{N_{tot}}$. Thus the main data input is a *T* × 3 matrix that lists the state vector *S*(*t*) = (*S*_1_(*t*), *S*_2_(*t*), *S*_3_(*t*)) each period *t* = 1, …, *T*. Each population state *S*(*t*) lies in the *simplex*, the two dimensional triangle $S=\{\left(s_{1},s_{2},s_{3}\right)\in R^{3}:s_{i}\geq 0,\sum_{i}s_{i}=1\}$.

Fitness *W*_*i*_(*t*) = *W*_*i*⋅_ ⋅ *S*(*t*) ≡ ∑_*j*_ *W*_*ij*_ *S*_*j*_(*t*) of morph *i* = 1, 2, 3 at time *t* is simply its weighted average fitness against current population frequency. Thus mean fitness at time *t* is $\bar{W}\left(t\right)=S\left(t\right)W\cdot S\left(t\right)\equiv S\left(t\right)\cdot \left(W_{1}\left(t\right),W_{2}\left(t\right),W_{3}\left(t\right)\right)\equiv \sum_{i}\sum_{j}W_{ij}S_{i}\left(t\right)S_{j}\left(t\right)$. Deterministic *replicator dynamics* (continuous time: [15]; discrete time: [2]) give the next period population state Z(t+1)∈S that would arise absent stochastic disturbances, and are specified by the difference equation

$$\begin{array}{c} Z_{i}\left(t+1\right)=\frac{W_{i}\left(t\right)}{\bar{W}\left(t\right)}S_{i}\left(t\right). \end{array}$$ (1)

Our model’s stochastic specification is novel in some respects but it is rooted in first principles. Here we sketch the main ideas; see S1 Text for details. The best known stochastic specification, adding a normally-distributed error to deterministic dynamics, unfortunately leads to nonsensical results for the simplex: the components of the state vector generally do not sum to 1.0 and may be negative. Truncations and renormalizations can push the state back into S, but then one no longer can rely on the nice properties of the normal distribution. The multinomial logit model is a viable alternative, but it is difficult to recover structural parameters from its estimated coefficients.

For the simplex S, the natural stochastic specification involves the Dirichlet distribution, *Dir*(*ηZ*) with parameters *η* and *Z* = (*z*_1_, *z*_2_, *z*_3_). This distribution has the following density function over $(x_{1},x_{2},x_{3})\in S$:

$$\begin{array}{c} f\left(x_{1},x_{2},x_{3}|\eta ,z_{1},z_{2},z_{3}\right)=K\left(\eta Z\right)x_{1}^{\eta z_{1}-1}x_{2}^{\eta z_{2}-1}x_{3}^{\eta z_{3}-1}I_{x\in S}, \end{array}$$ (2)

where *I* is the indicator function which is 0 outside the simplex and 1 on the simplex, and the normalizing constant *K*(*ηZ*) ensures that density integrates to 1. The intuition is as follows. Suppose that there is an urn with an infinite number of orange, blue and yellow balls of unknown proportions, and we observe a finite sample of *η* > 0 independent random draws from that urn. If the fractions of orange, blue, and yellow balls in that sample are *Z* = (*z*_1_, *z*_2_, *z*_3_), then the likelihood that the actual fractions in the urn were (*x*_1_, *x*_2_, *x*_3_) is given by density function (2). In technical jargon, *Z* has a multinomial distribution and the Dirichlet is the conjugate prior distribution.

Thus we complete the structural model via *S*(*t* + 1) ∼ *Dir*(*ηZ*(*t* + 1)), where *η* > 0 is the effective sample size of breeding adult males. That is, the deterministic replicator dynamics would give the next state precisely if the lizard population sizes were infinite, but given sampling error the distribution of a finite size population has a particular distribution. By well-known properties of the Dirichlet distribution (e.g., Kotz *et al.* 2000), we can say that *S*_*i*_(*t* + 1), the next generation fraction of morph *i*, is a random variable with mean *Z*_*i*_(*t* + 1) as specified in the deterministic replicator model, and with variance $\frac{Z_{i}\left(t+1\right)\left[1-Z_{i}\left(t+1\right)\right]}{\eta +1}$.

Mathematical and biological principles constrain the parameters we seek to estimate. Each fitness matrix entry *W*_*ij*_ ≥ 0 because, even when most prevalent, a morph *j* at worst can drive fitness (and thus next period’s share) of morph *i* to zero; negative shares are not possible. Stochastic disturbances are constrained to keep the state vector within the simplex, but the Dirichlet distribution automatically enforces that constraint. Since matrix entries appear in both the numerator and denominator of Eq (1), there is no effect when we multiply all matrix entries by an arbitrary constant *c* > 0. Hence we need to normalize the matrix, and do so via the constraint ∑_*i*_∑_*j*_ *W*_*ij*_ = 1, i.e., we normalize the original matrix by multiplying by $c=\frac{1}{\sum_{i}\sum_{j}W_{ij}}$.

Additionally, we can augment the regression with covariates that capture time- or world-specific shifts in the fitnesses. Probably the most important time-specific covariate is temperature, for which we use $\tau_{k}\left(t\right)=h_{r}\left(t\right)-{\bar{h}}_{r}^{k}$, a measure of temperature restriction the previous summer (when progeny are recruiting) normalized for each world *k* by subtracting the historical mean value ${\bar{h}}_{r}^{k}$ over the life of that world. For world-specific shifts we take *ν*_*k*_(*t*), the male population size in world *k* in year *t*, normalized by subtracting its world-specific mean value. We assume that these covariates shift fitness multiplicatively, with the proportional change in fitness coefficient a linear function of each variable. Thus we will estimate the generously parameterized fitness matrices Ω_*k*_ = ((Ω_*ijk*_))_*i*,*j*=1,…,3_ where

$$\begin{array}{c} \Omega_{ijk}\left(t\right)=W_{ij}\exp(\beta_{ij}^{\nu }\cdot \nu_{k}\left(t\right)+\beta_{ij}^{\tau }\cdot \tau_{k}\left(t\right)) \end{array}$$ (3)

The normalization of *ν* and *τ* ensures that we have Ω_*k*_(*t*) = *W* when both variables are at their average levels. In the most complete specification, therefore, in addition to the 3×3 fitness matrix *W* we are also estimating two other 3 × 3 coefficient matrices: *β*^*ν*^ for population density effects and *β*^*τ*^ for temperature effects.

Substituting Ω_*k*_ for *W* in Eq (1), one sees again that the *β* matrices need normalization. A natural choice is that the coefficients sum to zero. Thus estimation of Eq (3) is subject to the restrictions $W_{ij}\geq 0,\;\;\;\sum_{i,j}W_{ij}=1,\;\;\;\sum_{i,j}\beta_{ij}^{\nu }=0,\;\;\;\sum_{i,j}\beta_{ij}^{\tau }=0.$

Ignoring the covariates for the moment, we seek to estimate parameter vector

*θ* = (*η*, *W*_11_, *W*_12_, …, *W*_33_), under the maintained assumption that the observed state *S*(*t*) has the distribution *Dir*(*ηZ*(*t*)), where *Z*(*t*) is the row vector of 3x3 deterministic replicator dynamics. Thus $Z_{i}\left(t\right)=\frac{W_{i}\left(t-1\right)}{\bar{W}\left(t-1\right)}S_{i}\left(t-1\right)$. Given the previous state *S*(*t* − 1), the conditional density of the current state is

$$\begin{array}{c} f\left(S\left(t\right)|S\left(t-1\right)\right)=\frac{\Gamma (\eta )}{\prod_{i=1}^{3}\Gamma (\eta Z_{i}\left(t\right))}\prod_{i=1}^{3}S_{i}{\left(t\right)}^{\eta Z_{i}\left(t\right)-1}. \end{array}$$ (4)

This expression spells out the normalizing constant *K*(*ηZ*) in Eq (2), using the Gamma function $\Gamma \left(z\right)\equiv \int_{0}^{\infty }y^{z-1}e^{-y}dy.$

Summing the log of the right hand side of Eq (4) over *t* = 1, …, *T*, the conditional log-likelihood function of the sample is:

$$\begin{array}{c} \ln\ell =T\ln\Gamma \left(\eta \right)+\sum_{t=1}^{T}\sum_{i=1}^{3}[-\ln\Gamma \left(\eta Z_{i}\left(t\right)\right)+\ln S_{i}\left(t\right)\left(\eta Z_{i}\left(t\right)-1\right)] \end{array}$$ (5)

The *t* = 1 terms do not contribute to the sum since, with no previous period to which to apply replicator dynamics, their likelihood is 1. We maximized the right hand side of Eq (5) numerically over the set of parameter values *θ* = (*η*, *W*_11_, *W*_12_, …, *W*_33_) satisfying *η* > 0, *W*_*ij*_ ≥ 0, and $\sum_{i,j=1}^{3}W_{ij}=1$. The numerical maximization is via Matlab’s active set algorithm fmincon, using as initial values 1/9 for each entry *W*_*ij*_ of the payoff matrix and 20 for *η*. The algorithm converged reasonably quickly to the values reported in the Results section for a range of other initial values, e.g., for *η* between 15 and 30. The Matlab code is in S1 Code.

Extending the basic model to include covariates and panel data is conceptually straightforward. To deal with 4 separate locations (“worlds”) we write the conditional log-likelihood function for location *k* as:

$$\begin{array}{c} \ln\ell_{k}=T_{k}\ln\Gamma \left(\eta \right)+\sum_{t=1}^{T_{k}}\sum_{i=1}^{3}[-\ln\Gamma \left(\eta Z_{ik}\left(t\right)\right)+\ln S_{ik}\left(t\right)\left(\eta Z_{ik}\left(t\right)-1\right)] \end{array}$$ (6)

Note that *η* and the *W*_*ij*_’s are not location-specific because, to keep the number of free parameters small relative to overall sample size, we assume that the underlying stochastic structural model is the same in all locations. Of course stochastic realizations can and do vary across locations. Adding the covariates *ν* and *τ* affects the likelihood function only through the (deterministic) replicator dynamic equation for *Z*_*ik*_(*t*), where the payoff matrix *W* is replaced by the augmented fitness matrix Ω_*k*_(*t*) defined in Eq (2) of the text.

We now assume that stochastic realizations are conditionally independent, i.e., conditional on *S*_*ik*_(*t* − 1) and other covariates, the *S*_*ik*_(*t*)’s are independent across *k*. Then the augmented model has log-likelihood function

$$\begin{array}{c} \sum_{k\in \{MW,OW,BW,YW\}}\ln\ell_{k}, \end{array}$$ (7)

where ln *ℓ*_*k*_ is defined in Eq (6) using the augmented fitness matrices Ω_*k*_ in the replicator expression defining *Z*_*ik*_(*t*). In addition to the coefficient constraints on *W* mentioned earlier, we also imposed $\sum_{i,j}\beta_{ij}^{\nu }=0,\sum_{i,j}\beta_{ij}^{\tau }=0$, and used the agnostic initial values $\beta_{ij}^{\nu }=\beta_{ij}^{\tau }=0$. To avoid regions of the parameter space where the algorithm failed to converge we also imposed $\beta_{ij}^{\nu },\beta_{ij}^{\tau }\in \left[-10,10\right]$ in the primary replicator model (and $\beta_{ij}^{\nu },\beta_{ij}^{\tau }\in \left[-200,200\right]$ in the diploid sexual model mentioned in the next paragraph), but these constraints never bind once the algorithm approaches convergence.

S1 Text discusses a more complex diploid sexual model. It employs a fairly general expression for diploid genotype selection and transmission adapted from [16], [17] (Chapters 5 and 14), [13] and [18]. We consider a purely additive genetic model of transmission, which is most analogous to the simple replicator described above.

The discussion below of dynamic implications presumes several properties of equilibria, e.g., that the payoff matrix ${\hat{\Omega }}_{t}^{k}\equiv \Omega_{k}{|}_{\tau_{k}\left(t\right),\nu_{k}\left(t\right)}$ has a unique stable interior equilibrium when the temperature and density covariates (*τ*_*k*_(*t*), *ν*_*k*_(*t*)) are sufficiently close to zero. That discussion is justified by three known analytic results (see, for example, [19]) about equilibria of symmetric games specified by an arbitrary *n* × *n* payoff matrix *W*: (a) If it lies in the interior of the simplex, the vector S* = *cW*^−1^**1** is a (mixed) Nash equilibrium, where **1** is the vector of 1’s and the normalizing constant satisfies *c*^−1^ = **1** *W*^−1^ ⋅ **1**. Moreover, if the payoff matrix has cyclic best responses and each action is its own worst response, then (b) S* is the only NE and it indeed lies in the simplex interior, and (c) S* is also an ESS, hence dynamically stable.

## Results

**Fig 2:**
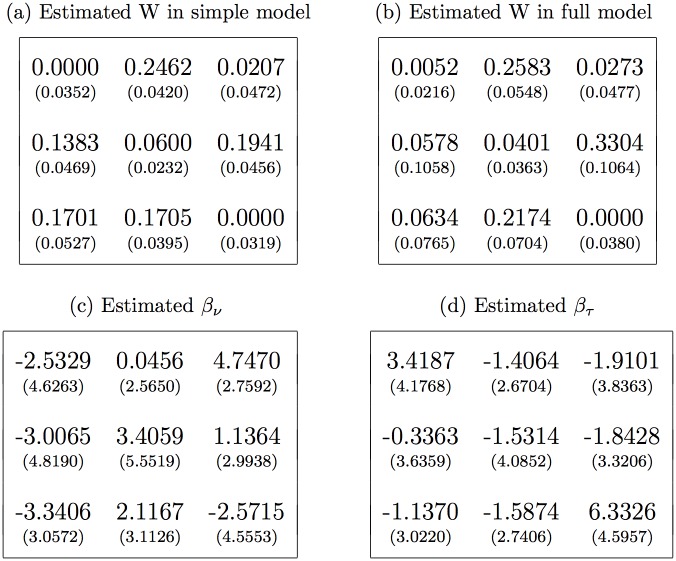
Estimates of the (*W*, *β*_*ν*_, *β*_*τ*_) model (with bootstrapped standard errors in parentheses). Matrix entries are for strategies [1, 2, 3] = [*o*, *b*, *y*]. In Panel (a) the covariates are suppressed, and the estimated effective sample size is $\hat{\eta }=21.7\pm 3.4$. The remaining panels show estimates of the full model, for which $\hat{\eta }=34.2\pm 1.2$.

Fig 2A presents the basic ML estimates, which assume that the same fitness matrix applies each year in each world. Note that (to a good degree of confidence) the highest entry in column 1 is *W*_13_ ≈ 0.17, indicating that strategy 3 (yellow, sneaker) is the best reply to strategy 1 (orange, aggressive). Likewise the data confirm that strategy 2 (blue, bourgeois) is the best reply to 3, and that strategy 1 is the best reply to 2. Thus estimates confirm the RPS best reply cycle. However, the estimated matrix is what [6] call an apostatic RPS (rather than pure RPS) in that each strategy is its own worst reply.

A more complete model for the replicator allows covariates to amplify (or diminish) each payoff matrix entry. A priori, the most important covariates appear to be population density that year (relative to its time average level for that world), and temperature as captured in a measure of excessive heat during the key time period. ML fits of this more complete model appear in Fig 2B, 2C and 2D. The apostatic RPS structure remains after including the covariates, while the constraint *W*_*oo*_ ≥ 0 no longer binds, perhaps due to negative effect that higher temperatures have on orange when prevalent. The main density effects are (a) negative for all morphs when orange is prevalent, as would be expected for the despotic orange strategy [20], and (b) positive for orange when yellow is prevalent, possibly indicating a fitness boost for orange when it is rare and conditions are conducive to a large population. The strongest temperature effect is a boost to yellow when it is prevalent. Covariates are jointly highly significant according to the standard likelihood ratio test. The main results hold up under a variety of robustness checks (Table M in S1 Text).

As a final robustness check we also estimated two versions of the diploid sexual model, one using male genotype frequencies as data for both male and female genotypes, and the other using observed male and female genotype frequencies. Analysis of the diploid sexual model, restricting analysis to the male genotype frequencies, recovers similar payoffs as observed in the replicator model (Table Q in S1 Text), and inclusion of female genotype frequencies, additionally recovers a component of the blue enhancing its own fitness via cooperation (Table R in S1 Text), a feature of the known dynamics of the *Uta* RPS [21], [10].

Returning to the featured replicator model, the estimated matrices have implications for dynamic behavior. If the temperature and density covariates were always fixed at their average levels, then the estimates in Fig 2B imply that from any interior initial condition (including the large experimental perturbations to OW, BW and YW) the state would converge asymptotically and quite rapidly to the equilibrium S* = λ*W*^−1^**1** ≈ (0.38, 0.40, 0.22), via a counterclockwise swirl. Even for fairly extreme initial conditions, near a corner of the simplex but with each morph share at least 0.1, it takes at most 5-8 years for the state to converge within sampling error to S*.

**Fig 3:**
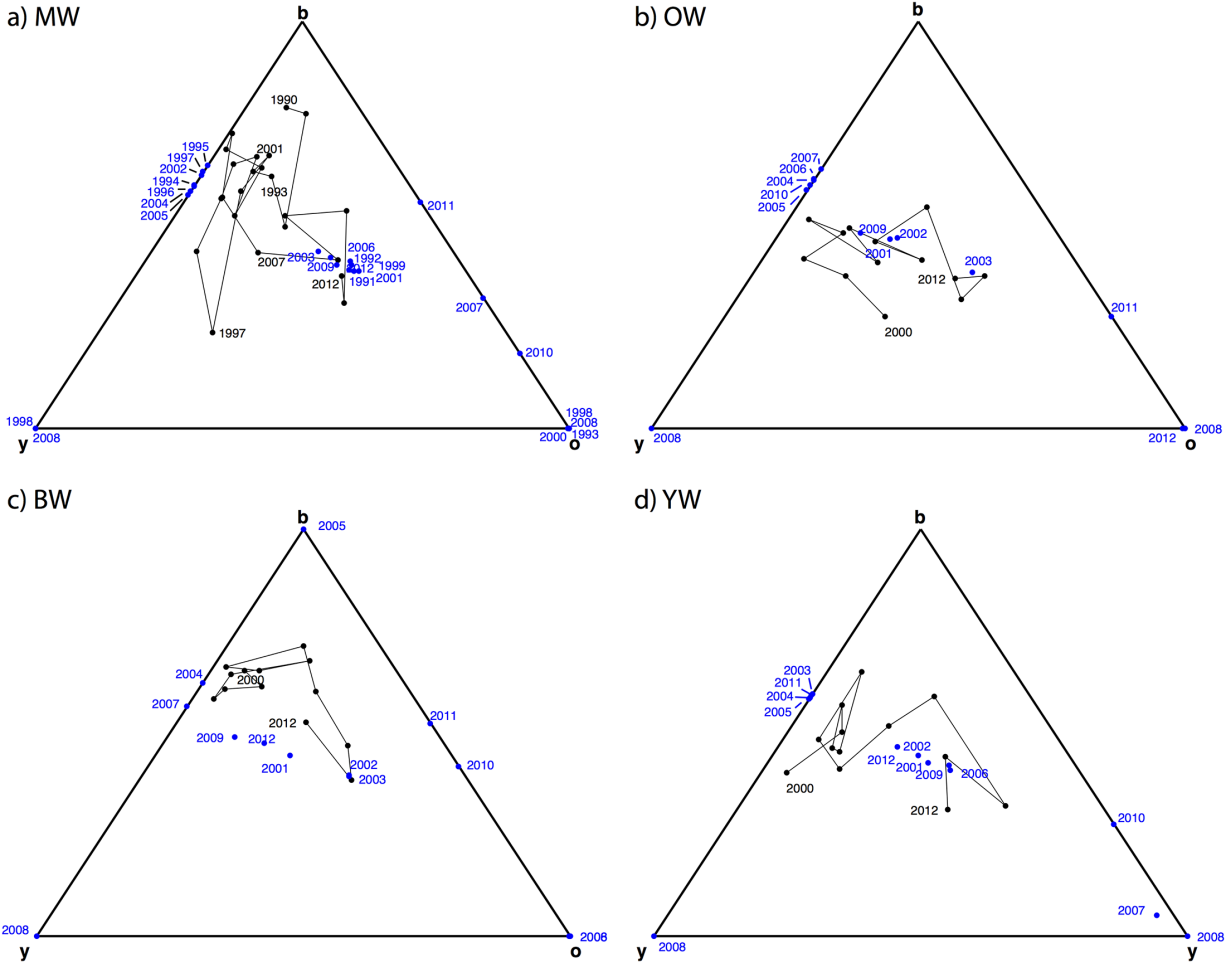
Simplex and states in all worlds. Black points and lines are time series *S*(*t*), while blue points are the Nash equilibrium for ${\hat{\Omega }}_{t}^{xW}$.

Of course, covariates are not fixed, and indeed vary stochastically from year to year and across locations. Climate directly influences morph payoffs via progeny recruitment during hot versus cool summers, and population density has an indirect influence. To assess the impact, we compute the effective payoff matrix ${\hat{\Omega }}_{t}^{k}$ for each World (*k* = *MW*, *OW*, *BW*, *YW*) and each year *t* by amplifying or diminishing each payoff entry according to the density and temperature variables experienced in that world that year. The blue dots in Fig 3 show drastic year-to-year changes in the MW equilibrium, i.e., the Nash equilibrium (here, also the ESS) for the payoff matrix ${\hat{\Omega }}_{t}^{M}$. In many years the equilibrium lies on an edge, indicating, under the conditions experienced that year, that one morph (the one fixated at the corner of the simplex opposite that edge) is in danger of local extinction.

**Fig 4:**
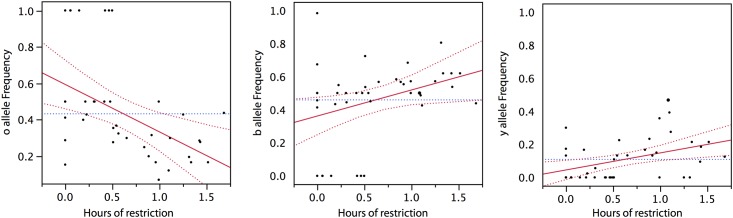
Geographic variation in the orange, blue and yellow strategy types and hours of restriction during juvenile emergence.

We tested the proposition that climate can fix the game on an edge using published phylogeographic data on morph frequency collected across the range of the side-blotched lizard in the United States and Mexico, in which we have observed 7 independent losses of the yellow allele and two losses of the blue allele [22]. We estimated hours of temperature restriction (see S1 Text), *h*_*r*_, using maximum daily air temperature, *T*_*max*_, obtained from worldclim.org, the relationship between monthly *T*_*max*_ and monthly *h*_*r*_ at Los Banos, published data on body temperature of *Uta*, *T*_*b*_, across its range, and a published physiological model of climate forcing relating *h*_*r*_ to the difference between *T*_*max*_ and *T*_*b*_ [7]. The predicted NE from our climate forcing model of the RPS game, when driven by *h*_*r*_ estimated in the local populations, accurately predicts observed frequency of o alleles (F-test: *F*_1,39_ = 17.87, *P* < 0.0001), b alleles (*F*_1,39_ = 22.78, *P* < 0.0001) and y alleles (*F*_1,39_ = 5.48, *P* < 0.03) (Fig 4). Correlations between observed and predicted frequency of o (*P* = 0.02) and b alleles (*P* = 0.003) are also significant using phylogenetic independent contrasts. Finally, the hours of restriction observed across the geographic range is negatively related to the observed frequency of orange (*F*_1,39_ = 9.98, *P* < 0.004) but positively related to blue color strategies (*F*_1,39_ = 5.98, *P* < 0.03), and yellow strategies (*F*_1,39_ = 8.21, *P* < 0.007), with the strategy yellow perilously close to fixation under extremely cool conditions of *h*_*r*_ found in northern populations or along the California Coast, where orange is likewise strongly favored.

## Discussion

To summarize, our first substantive finding is that the overall payoff matrix estimated for male side-blotched lizards from one generation to the next is of the type called apostatic RPS [6]: both rare strategies have higher fitness than the common strategy, and the best replies are intransitive. Using subscripts {*o*, *b*, *y*} instead of {1, 2, 3} to denote the orange, blue and yellow color morphs, the estimated fitness orderings are: *W*_*yo*_ > *W*_*bo*_ > *W*_*oo*_, *W*_*ob*_ > *W*_*yb*_ > *W*_*bb*_ and *W*_*by*_ > *W*_*oy*_ > *W*_*yy*_.

Previous estimates of the male lizards’ siring success using other methods [6] indicated a pure RPS type of payoff matrix: *W*_*yo*_ > *W*_*oo*_ > *W*_*bo*_, *W*_*ob*_ > *W*_*bb*_ > *W*_*yb*_ and *W*_*by*_ > *W*_*yy*_ > *W*_*oy*_. The discrepancy relative to the previous estimates consists of reduced fitness for common strategies. Indeed, our current estimates put all three common fitnesses (matrix entries along the main diagonal) near zero.

How might the discrepancy arise? Mindful that previous estimates use a narrower notion of fitness (siring success) than do current estimates (male offspring surviving to adulthood), we can think of two plausible sources of the discrepancy. First, male hatchlings of the common type may have lower first year survival rates, arising from strong selection against self genotypes exerted either by surviving adult males that are still present when progeny emerge or by the progeny themselves as they establish territories [23], [24]. Second, as argued by [16], females may prefer rare male genotypes [18], [25], and more frequently select their sperm [26], [6], chose them as mates [9], [27], incur incubation mortality due to mismatch of co-adapted gene complexes [24], or as suggested by [28], females may selectively produce female progeny when the common male strategy is disadvantaged and the rare strategies are advantaged in the progeny generation. Because adult male density is highly correlated with female density (*R*^2^ = 0.63, *t* − *value* = 10.38, *P* < 0.0001, removing effect of world) and frequency [8] (results in S1 Text), many of these factors noted above associated with female genotypes will be sequestered in the density covariate in our ML model.

The dynamic consequences of the new estimates deserve further comment. The picture emerging from previous estimates of a stable pure RPS matrix was of a gradual inward counterclockwise spiral perturbed a bit by noise. New estimates of an apostatic RPS instead imply that unperturbed dynamics are highly stable swirls and a full counterclockwise cycle would converge precisely to the stable mix of three morphs. However, our second substantive finding is that the covariates imply large perturbations in most years (indeed, the equilibrium is often located on an edge of the simplex, Fig 3), so the system is chasing (with a typically counterclockwise approach) an erratic moving target.

Besides a better qualitative account of dynamics in each world separately, the new picture can explain the observed approximate entrainment, in less than a decade, of the RPS cycle across the four “worlds,” including all three that were experimentally perturbed to be completely out of phase with the unperturbed control outcrop. Temperature, one of the two covariates considered, perturbs the dynamic path similarly in each world each year, and thus suggests entrainment.

This new picture of the evolutionary dynamics at a local scale also explains morph variation across the geographical range of the side-blotched lizard. In northern populations longterm climate variation pushes the NE close to the orange-blue edge of the simplex. Indeed, many northern populations in both the Great Basin and the Coast Range of California exhibit fixation on orange and orange-blue combinations, with yellow being the most susceptible to selective loss [22]. This loss likely arises because yellow sires derive most paternity fitness from later season clutches [26], but cooler and shorter seasons in northern populations removes the advantage of yellow males, fixing on orange and orange-blue combinations. This process of climate-forced allele fixation generates considerable mating system diversity, as strategies are lost the mating system collapses to ob or o states. However, large gradients in climate across the spatial scale of the entire geographic range of the species can drive such allele fixation. For example, the gradient in temperature from the Central Valley of the Coast Range of California has resulted in the fixation of a trimorphic lineage to a monomorphic lineage, and a similar change in gradient from NV to OR and UT has resulted in the fixation of a trimorphic lineage to two different monomorphic lineages. Other fixations associated with cooler climate are also found across the range [22].

One can imagine more sophisticated structural models of morph dynamics than considered here. We have treated population density as exogenous, but surely it is partly endogenous and a key aspect of female strategies [8]. One might also like to know more about different fitness components, in particular, those arising from female mate preference, male siring success, progeny survival, and sex ratio modulation [28]. S1 Text proposes a structural model of diploid replicator dynamics with multiplicative components that account for these fitness components. Beyond this, one can imagine structural models that incorporate nonlinearities assumed away when a matrix is used to model fitness payoffs. However, currently available *Uta* data comprise relatively short time series and a modest number of distinct populations, and thus are inadequate for estimating models with far more parameters than ours. The diploid sexual model does recover similar payoffs, when testing it with the same information as the replicator model (e.g., male only genotypes), and the sexual model using male and female genotypes additionally recovers aspects of the male game of cooperation involving blue alleles, also key aspects of the *Uta* RPS reported in previous studies [21], [12]. Female *Uta* also carry the same genes for finding self-similar genotypes, but instead of using them to enhance cooperation, they are used in mate preference of blue females for blue males [12].

The techniques introduced here do seem widely applicable when the concern is overall fitness rather than its components and when frequency dependent selection can be captured in a payoff matrix. Inviting applications include bacterial RPS systems [29] and Y-linked RPS systems in fishes [30]. Our techniques can easily be specialized to two-morph systems such as the spadefoot toad polyphenism [31], [32] when there are experimental or natural perturbations that push the system away from equilibrium. In general, two strategy one-population games are unlikely to cycle unless they involve density regulation, as with the r-K strategies of *Uta* females noted earlier, or mammalian Chitty cycles [33]. Extensions of our techniques to more than three morphs (e.g., five as in *Poecilia parae*; see Bourne *et al.* 2003) in a single population seem straightforward, and we see no obstacle to applying them to frequency dependent selection in two or more interacting populations such as lynx-hare cycles [34], competition cycling dynamics [35], or host-parasite cycling dynamics [36]. Given the analogous processes maintaining diversity in mating systems and ecosystems [6], [35], our approach highlights the role of climate in potentially reducing ecosystem diversity at small spatial scales, but enhancing diversity at large spatial scales.

## Supporting information

S1 Text (PDF)

S1 Data (CSV)

S1 Code (ZIP)
