# Supplementary material for: Evolutionary games, climate and the generation of diversity
Source: PLoS One. 2017 Aug 31;12(8):e0184052. doi: 10.1371/journal.pone.0184052 (PMC5578633; doi:10.1371/journal.pone.0184052)
Supplement: S1 Text — (PDF) [file pone.0184052.s001.pdf]

Supporting Information for Evolutionary Games, Climate and the Generation of Diversity

Daniel Friedman *

Economics Department, UC Santa Cruz

Jacopo Magnani †

Division of Social Science, New York University Abu Dhabi

Dhanashree Paranjpe ‡

Department of Ecology and Evolutionary Biology, UC Santa Cruz

Barry Sinervo §

Department of Ecology and Evolutionary Biology, UC Santa Cruz

August 24, 2017

*dan@ucsc.edu, leeps.ucsc.edu

†jacopo.magnani@gmail.com

‡dhanu@ucsc.edu

§lizardrps@gmail.com

Stochastic Structure. Any statistical procedure to estimate a parameter vector θ must specify a general form $G(x, \theta, e)$ for the relevant variables x (here, morph frequencies, which we refer to as shares, and covariates), including a stochastic structure for error e to account for data variability y not fully captured in the deterministic component $G(x, \theta, 0)$.

The best known error structure is Normal-additive: the investigator finds the vector θ that best fits the equation $y_t = G(x_t, \theta, 0) + e_t$, e.g., by minimizing residual sum of squares $\hat{e}_t = y_t - G(x_t, \theta, 0)$. One assumes that $G(x_t, \theta, 0)$ is independent of the random variable e_t, with mean zero and a Normal distribution. This error model has the advantage of familiarity and ease of interpretation. For the share data to which standard evolutionary models apply, however, it has a severe disadvantage – it doesn't respect the simplex constraints, and so can predict negative shares and other nonsensical behavior.

For fitting share or proportional data, then, we need an error model that obeys simplex constraints that (a) each component of $G(x, \theta, e)$ is nonnegative and (b) they sum to 1. The Logit error model is also widely known and automatically satisfies (a). As explained later, it can be generalized and normalized so that it also satisfies (b). Its main disadvantage is that, as explained below, the estimated coefficients don't quite correspond to the parameters of interest, i.e., estimates are not statistically consistent and can be difficult to interpret.

The Dirichlet error model is less well known, but by construction it satisfies (a) and (b), delivers consistent estimates of θ, and can be extended to deal with interactive covariates.

Simulation Exercise. To check that the estimation procedure is consistent and robust, we use the Dirichlet-Replicator model with various parameters to generate artificial data samples of a size similar to the field data. We then checked whether the estimation procedure could recover the specific parameters chosen to generate the data.

Our first such exercise sets the parameter $\eta = 25$ and sets W as in Panel (a) of Table A below, an apostatic RPS matrix with interior NE at $(1/3, 1/3, 1/3)$. Apply the Dirichlet-Replicator model to generate sample time series of 60 periods each. The average estimated payoff matrix over 50 such samples appears in Panel (b) of the table. Somewhat reassuringly, the average fitted matrix is also an apostatic RPS, and its coefficients are all reasonably close to the true values used in the simulation.

A very tough example sets $\eta = 25$ and but now takes W as in Panel (a) of Table B below, a barely apostatic RPS matrix with interior NE at $(0.8, 0.1, 0.1)$ not far from a corner. Again we use the model to generate samples of length 60 and, in panel (b), report average estimates over 50 samples. The estimated W is almost (but not quite) apostatic RPS: the entries in the first column are biased downward and there is a virtual tie there for the best reply.

(a) True W

0.0300	0.2217	0.0816
0.0816	0.0300	0.2217
0.2217	0.0816	0.0300

(b) Estimated W

0.0303 (0.0371)	0.2411 (0.0517)	0.0657 (0.0422)
0.0853 (0.0381)	0.0357 (0.0344)	0.2113 (0.0416)
0.2242 (0.0489)	0.0898 (0.0485)	0.0165 (0.0286)

Table A: Symmetric apostatic RPS case. Dirichlet simulation uses true $\eta = 25$; estimated $\hat{\eta} = 28.42 \pm 3.98$.

(a) True W

0.0638	0.3493	0.0779
0.0779	0.0174	0.2119
0.0952	0.0779	0.0287

(b) Estimated W

0.0066 (0.0121)	0.2952 (0.1082)	0.1252 (0.0842)
0.0246 (0.0214)	0.0482 (0.0613)	0.2279 (0.1028)
0.0287 (0.0244)	0.1867 (0.1005)	0.0569 (0.0614)

Table B: Asymmetric case. True $\eta = 25$, estimated $\hat{\eta} = 33.60 \pm 5.03$.

A Logistic Alternative. The logistic regression model is well known for explaining a single variable with range [0, 1], such as the probability that a particular event occurs or does not occur. That regression model can be adapted for probability vectors over $m > 1$ possible outcomes (or share data for $m > 1$ morphs) as follows.

The generalized log-odds function g maps a point $s = (s_1, ..., s_m) \in \mathcal{S}^m$ to the point

$(\ln s_1, ..., \ln s_m) \in \mathbb{R}^m$ and then projects orthogonally onto the $m-1$ dimensional null subspace $\mathbb{R}^m_0$ — the vectors in $\mathbb{R}^m$ whose components sum to zero — by subtracting $\frac{1}{m}\sum_{j=1}^m \ln s_j$ from each component. The g function has a global inverse known as the multinomial logistic function, $L : (y_1, ..., y_m) \in \mathbb{R}^m_0 \mapsto (\frac{e^{y_1}}{\sum_j e^{y_j}}, ..., \frac{e^{y_n}}{\sum_j e^{y_j}}) \in \mathcal{S}^m$. In particular, for $m = 3$, the map $g : \mathcal{S}^3 \to \mathbb{R}^3_0$ is given by $s \mapsto \frac{1}{3}(\ln \frac{s_1^2}{s_2 s_3}, \ln \frac{s_2^2}{s_1 s_3}, \ln \frac{s_3^2}{s_1 s_2})$. To verify that $L^{-1} = g$ for $m = 3$, simply confirm that for $s_i = L_i(y_1, y_2, y_3) = \frac{e^{y_i}}{e^{y_1}+e^{y_2}+e^{y_3}}$ we have $\frac{1}{3}\ln \frac{s_1^2}{s_2 s_3} = \frac{1}{3}\ln e^{3y_1 - 0} = y_1$ if $y_1 + y_2 + y_3 = 0$, and similarly for y_2 and y_3.

The generalized Logistic regression model applies the logistic function to the dependent variable y_t, which in our application is the current state vector $S(t) = (s_1, s_2, s_3) \in \mathcal{S}$. Thus the left hand side variable in the regression will be

$$Y(t) = g(S(t)) = \frac{1}{3}(\ln \frac{s_1^2}{s_2 s_3}, \ln \frac{s_2^2}{s_1 s_3}, \ln \frac{s_3^2}{s_1 s_2}) \tag{1}$$

Likewise, the main explanatory variable will be the generalized log odds of the replicator prediction $Z(t) = (z_1, z_2, z_3) \in \mathcal{S}$, so

$$X(t) = g(Z(t)) = \frac{1}{3}(\ln \frac{z_1^2}{z_2 z_3}, \ln \frac{z_2^2}{z_1 z_3}, \ln \frac{z_3^2}{z_1 z_2}), \tag{2}$$

where, $Z(t)$ is obtained for $t > 1$ from $S(t-1)$ using the discrete replicator equation.

By virtue of the g transformation, the covariates ν and τ for density and temperature restriction can simply be added to the right hand side of the equation and we can assume an additive normal error. Thus the equation we will estimate is

$$Y_i(t) = X_i(t) + \beta_i^\nu \nu_t + \beta_i^\tau \tau_t + \epsilon_i(t), \tag{3}$$

$$\epsilon_i(t) \sim N(0, \sigma_i^2), \qquad i = 1, 2, 3. \tag{4}$$

The parameters of the model are the payoff matrix W, the six covariate sensitivities β_i^τ and β_i^ν, and the three noise variances σ_i^2.

Since $\epsilon_i(t) \sim N(0, \sigma_i^2)$ the conditional density of an observation $Y(t)$ (simplifying expressions by setting the β's to zero for the moment) is given by:

$$f(Y(t)|Y(t-1)) = \prod_{i=1}^{3} \frac{1}{\sqrt{2\pi\sigma_i^2}} \exp\left\{ -\frac{[Y_i(t) - X_i(t)]^2}{\sigma_i^2} \right\} \tag{5}$$

Taking products over the sample of observations and then taking logs, we obtain the log-likelihood function

$$\ln \ell = \sum_{t=1}^{T} \sum_{i=1,2} -\frac{1}{2} \ln 2\pi - \ln \sigma_i - \frac{[Y_i(t) - X_i(t)]^2}{\sigma_i^2}. \tag{6}$$

To save parameters (without doing any apparent violence to our data) we assume that all three σ_i's have the same value, which we refer to as the error amplitude. Of course, when there are covariates such as ν and τ, one includes with $X_i(t)$ the other systematic terms on the right hand side of (3). We again use the Matlab routine `fmincon` for the numerical optimization, and again impose the usual coefficient constraints and starting values.

The logistic normal model keeps the likelihood of the data very tractable and at the same time restricts the variables to the simplex. However, note that a structural interpretation of the coefficients is compromised. To explain, for notational simplicity suppose again that the covariates are known to be zero. Although $E_{t-1}(Y_i(t)) = X_i(t)$, in general $E_{t-1}(S_i(t)) \neq Z_i(t)$ since $S = L(Y), Z = L(X)$ and L is nonlinear. That is, the maximum likelihood parameter estimates in (6) are biased. In some cases the bias is small but in others it is large, and it is hard to assess a priori.

Table C reports payoffs estimated by logistic regression with no covariates applied to allele panel data. It confirms the apostatic RPS structure obtained using the Dirichlet model.

0.1798<br>(0.0570)	0.0447<br>(0.1318)	0.0380<br>(0.0538)
0.3141<br>(0.1146)	0.0000<br>(0.1388)	0.0500<br>(0.1262)
0.3547<br>(0.1440)	0.0188<br>(0.1097)	0.0000<br>(0.0665)

Table C: Logistic estimates of W (error amplitude, $\hat{\sigma} = 0.41 \pm 0.36$.)

For completeness, Table D reports logistic estimates with covariates. Note that the covariates are not interactive, but rather are assumed to affect the level of fitness of each morph independent of the frequency of other morphs. It turns out that none of the covariate sensitivity estimates is significantly different from zero.

(a) Estimated W

0.1689	0.0666	0.0176
(0.0753)	(0.1099)	(0.0479)
0.3149	0.0000	0.0555
(0.1079)	(0.1021)	(0.0896)
0.3517	0.0249	0.0000
(0.1265)	(0.0830)	(0.0349)

(b) Estimated β_ν

-0.0851
(10.1634)
0.0303
(14.8639)
0.0549
(15.1466)

(c) Estimated β_τ

0.1394
(11.2624)
-0.0007
(8.0081)
-0.1387
(9.5621)

Table D: Estimates of the $(W, \beta_\nu, \beta_\tau)$ logistic model (Error amplitude, $\hat{\sigma} = 0.40 \pm 0.14$).

Can the Logistic regression model recover parameters used in the simulated data? We see from Tables E and F that it does a decent job in the easy symmetric case and even in the difficult asymmetric case the estimates bear some resemblance to the true parameters.

(a) True W

0.0300	0.2217	0.0816
0.0816	0.0300	0.2217
0.2217	0.0816	0.0300

(b) Estimated W

0.0536	0.2402	0.0628
(0.0540)	(0.1112)	(0.0560)
0.0697	0.0563	0.2017
(0.0522)	(0.0559)	(0.0878)
0.1908	0.0860	0.0388
(0.0900)	(0.0632)	(0.0447)

Table E: Logit estimation with symmetric simulated data

(a) True W

0.0638	0.3493	0.0779
0.0779	0.0174	0.2119
0.0952	0.0779	0.0287

(b) Estimated W

0.0921	0.2270	0.0919
(0.1040)	(0.1640)	(0.0790)
0.1033	0.0362	0.1540
(0.1027)	(0.0482)	(0.0964)
0.1259	0.1252	0.0444
(0.1140)	(0.1200)	(0.0523)

Table F: Logit estimation with asymmetric simulated data

Estimation of a non-linear VAR model. Sometimes researchers use linear models estimated via least squares to get a quick estimate of an event probability, or of marginal effects on an event probability, and then compare the results to those obtained with a proper model, such as logit, which respects the bounded range of the variables but is more complicated to set up and interpret. In that spirit, we report estimates of the payoff matrix and covariate sensitivities obtained by a linear regression model applied to our lizard data.

The basic linear model is

$$S_1(t) = \frac{W_{1,\cdot}S(t-1)}{S(t-1)'WS(t-1)} S_1(t-1) + \epsilon_1(t) \tag{7}$$

$$S_2(t) = \frac{W_{2,\cdot}S(t-1)}{S(t-1)'WS(t-1)} S_2(t-1) + \epsilon_2(t) \tag{8}$$

$$S_3(t) = 1 - S_1(t) - S_2(t) \tag{9}$$

$$\epsilon_1(t) \sim N(0, \sigma_1^2)$$

$$\epsilon_2(t) \sim N(0, \sigma_2^2)$$

where $W_{i,\cdot}$ is the i-th row of W. This model assumes Gaussian additive noise as in the previous section. Condition (9) guarantees that the estimated shares sum to one. On the other hand, this model does not guarantee that $S_i \in [0,1]$, although this condition is likely to be satisfied for sufficiently small noise variances.

The parameters of the model are the payoff matrix W and the noise variances (σ_1^2 and σ_2^2). As usual, set $Z_i(t+1) = \frac{W_i(t)}{\bar{W}(t)} S_i(t)$, the prediction from deterministic replicator dynamics. Thus $\epsilon_i(t) = S_i(t) - Z_i(t)$. Since $\epsilon_i(t) \sim N(0, \sigma_i^2)$ the conditional density of an observation $S(t)$ is given by:

$$f(S(t)|S(t-1)) = \prod_{i=1,2} \frac{1}{\sqrt{2\pi\sigma_i^2}} \exp\left\{ -\frac{[S_i(t) - Z_i(t)]^2}{\sigma_i^2} \right\}. \tag{10}$$

Taking products and logs as usual (or logs and sums), we obtain the log-likelihood function

$$\ln \ell = \sum_{t=1}^{T} \sum_{i=1,2} -\frac{1}{2} \ln 2\pi - \ln \sigma_i - \frac{[S_i(t) - Z_i(t)]^2}{\sigma_i^2}. \tag{11}$$

A simplifying assumption is that $\sigma_1 = \sigma_2 = \sigma$. Then maximizing the log-likelihood function with respect to entries of W is equivalent to the simpler non-linear least squares problem:

$$\min_W \sum_{t=1}^{T} \sum_{i=1,2} [S_i(t) - Z_i(t)]^2 \tag{12}$$

We solve this least squares minimization problem numerically using the Matlab routine `fmincon`, after imposing the usual non-negative entries and overall sum constraints on W. The algorithm converges quickly from neutral initial conditions and returns the estimated payoff matrices in Tables G and H.

0.0000<br>(0.0199)	0.2690<br>(0.0465)	0.0000<br>(0.0517)
0.1128<br>(0.0377)	0.0607<br>(0.0297)	0.2331<br>(0.0434)
0.0966<br>(0.0595)	0.1733<br>(0.0547)	0.0545<br>(0.0436)

Table G: Estimate of payoff matrix W using the basic VAR model. $\hat{\sigma} = 0.09 \pm 0.01$.

(a) Estimated W

0.0000<br>(0.0175)	0.2651<br>(0.0374)	0.0000<br>(0.0410)
0.1143<br>(0.0362)	0.0514<br>(0.0255)	0.2465<br>(0.0433)
0.0947<br>(0.0577)	0.1698<br>(0.0520)	0.0582<br>(0.0388)

(b) Estimated β_ν

-0.0110<br>(0.0179)
0.0110<br>(0.0179)

(c) Estimated β_τ

0.0176<br>(0.0285)
-0.0176<br>(0.0285)

Table H: Estimates of $(W, \beta_\nu, \beta_\tau)$ in the VAR model with covariates. $\hat{\sigma} = 0.40 \pm 0.14$.

Applied to the simulated data, we obtain the estimates reported in Tables I and J.

(a) True W

0.0300	0.2217	0.0816
0.0816	0.0300	0.2217
0.2217	0.0816	0.0300

(b) Estimated W

0.0318<br>(0.0383)	0.2417<br>(0.0540)	0.0631<br>(0.0460)
0.0864<br>(0.0422)	0.0351<br>(0.0372)	0.2107<br>(0.0425)
0.2243<br>(0.0505)	0.0892<br>(0.0500)	0.0177<br>(0.0256)

Table I: Symmetric case

(a) True W

0.0638	0.3493	0.0779
0.0779	0.0174	0.2119
0.0952	0.0779	0.0287

(b) Estimated W

0.0212 (0.0223)	0.3921 (0.1093)	0.0826 (0.0765)
0.0488 (0.0337)	0.0680 (0.0910)	0.1626 (0.1196)
0.0502 (0.0357)	0.1354 (0.1036)	0.0390 (0.0639)

Table J: Asymmetric case

Level Effects and Joint Significance Tests. Returning to the Dirichlet/MLE model, one can include level effects. To investigate, write the enhanced payoff matrix entries as

$$\Omega_{ijk}(t) = W_{ij} \exp\left(\beta^{\nu}_{i0} \cdot \nu_k(t) + \beta^{\tau}_{i0} \cdot \tau_k(t) + \beta^{\nu}_{ij} \cdot \nu_k(t) + \beta^{\tau}_{ij} \cdot \tau_k(t)\right). \tag{13}$$

The coefficient vectors $(\beta^{\nu}_{10}, \beta^{\nu}_{20}, \beta^{\nu}_{30})$ and $(\beta^{\tau}_{10}, \beta^{\tau}_{20}, \beta^{\tau}_{30})$ capture level effects, while as before matrices $((\beta^{\nu}_{ij}))$ and $((\beta^{\tau}_{ij}))$ capture interactive effects for the usual covariates (Table K).

(a) Estimated W

0.0006 (0.0107)	0.3155 (0.0397)	0.0101 (0.0327)
0.0236 (0.0198)	0.0579 (0.0212)	0.3054 (0.0441)
0.0634 (0.0537)	0.2113 (0.0432)	0.0121 (0.0239)

(b) Estimated β^{ν}_0

2.0135 (1.1219)
-0.3124 (0.8049)
-1.7011 (0.8371)

(c) Estimated β^{τ}_0

0.5595 (0.9181)
-0.5063 (0.7414)
-0.0532 (0.7610)

(d) Estimated β_{ν}

2.2313 (3.0258)	-2.8754 (1.4303)	1.2631 (2.2876)
-1.8104 (0.8562)	2.1362 (1.0255)	-0.7495 (0.7783)
-1.9771 (1.5562)	2.4074 (1.1605)	-0.6256 (1.7598)

(e) Estimated β_{τ}

-1.2542 (2.6133)	-0.0165 (1.1048)	2.4501 (1.5495)
-2.9744 (1.0563)	1.5900 (0.8457)	1.4670 (0.8467)
1.1492 (0.9420)	0.8879 (1.0734)	-3.2992 (2.0048)

Table K: Estimates of the $(W, \beta_{\nu}, \beta_{\tau}, \beta^{\nu}_0, \beta^{\tau}_0)$ Dirichlet model. Estimated effective sample size is $\hat{\eta} = 34.2 \pm 1.5$.

If significant, level effects would imply differences in the impact of covariates on payoffs, among populations, which would affect the location of the interior attractor. Note that none of the level effects is highly significant individually and only two are marginally significant. We will soon see (in the last line of Table L below) that the level effects are jointly insignificant given the interactive effects. Note that the estimated W matrix is again apostatic RPS. Table L below shows the statistical significance of the covariates as a whole. It reports results of the standard test, called likelihood ratio, for parametric restrictions in maximum likelihood estimation. This test compares the log likelihood of a restricted model that eliminates d degrees of freedom in a broader model. Under the null hypothesis, H0, that the restriction is valid within the broader model specified in the alternative hypothesis H1, the difference in log likelihoods (times 2) is distributed $\chi^2(d)$; see for example Davidson & MacKinnon (2004). The first row of the table shows that we can reject the null hypothesis that population density effects are insignificant with great confidence ($p < 0.001$). The second and third rows shows that temperature effects are marginally significant ($p < 0.10$, barely) by themselves, and that the combination is highly significant ($p < 0.0001$). The last row rejects level effects, i.e., the hypothesis that the covariates operate directly on the fitness of each morph, rather than interactively via all other morphs. Other robustness checks, e.g., showing that the fit is not appreciably improved by setting to zero the β coefficients that are not significant at the 5% level, are omitted to save space.

Table L: LR tests

H_0	H_1	Reject H_0 at 5%?	p-value	χ^2
$\beta^\nu = 0$	W, β^ν	Yes	6.9166×10^{-4}	28.8322
$\beta^\tau = 0$	W, β^τ	No	0.0903	15.0233
$\beta^\nu = 0, \beta^\tau = 0$	W, β^ν, β^τ	Yes	3.4629×10^{-5}	52.2187
$\beta^\nu = 0, \beta^\tau = 0$	$W, \beta^\nu, \beta^\tau, \beta^\nu_0, \beta^\tau_0$	Yes	0.0011	41.9012

Diploid transmission of OBY. In diploid sexual models, one parent is female and the other male. Below, we write a fairly general expression for diploid genotype selection and transmission, develop its implications regarding phenotype payoffs as related to OBY

genotypes, and show the sense in which replicator dynamics as specified in the main text approximates diploid sexual dynamics. That general expression is adapted from Alonzo & Sinervo (2001) and Friedman & Sinervo (2016, Chapters 5 and 14), Sinervo (2001) and Sinervo *et al.* (2007).

The general expression uses multi-matrices, which are commonly used to specify payoffs in multi-player (or multi-population) games.[1] The multi-matrix $\mathbf{Q} = [\mathbf{Q^1}, ..., \mathbf{Q^K}]$ has entries that are K-vectors, namely $(([q^1_{ij}, q^2_{ij}, ..., q^K_{ij}]))$, where $\mathbf{Q^k} = ((q^k_{ij})), k = 1, ..., K$ are the separate matrices. The general expression uses Hadamard matrix multiplication. If $\mathbf{A} = ((a_{ij}))$ and $\mathbf{B} = ((b_{ij}))$ are both $n \times n$ matrices, then the Hadamard product $\mathbf{A} \circ \mathbf{B} = \mathbf{C}$ is defined as entry-by-entry multiplication, so $c_{ij} = a_{ij}b_{ij}$ for each i, j. Hadamard products are convenient to work with because they commute and are readily decomposed. The identity matrix for this operation is $\mathbf{1}_{n\times n} = ((1))$, i.e., all entries are 1, in contrast to the identity matrix for ordinary matrix multiplication, $\mathbf{I}_n$, whose off-diagonal entries are 0.

Let the multi-matrix $\mathbf{Q}$ summarize genotype transmission from parents to progeny; examples appear below. Let matrices $\mathbf{F}$ and $\mathbf{M}$ summarize fertility selection for female and male parents, let $\mathbf{A}$ and $\mathbf{B}$ summarize their respective sexual preferences, and let $\mathbf{s}^F$ and $\mathbf{s}^M$ denote phenotype shares in females and males respectively. Then, for the appropriate normalization factor $c > 0$, the general expression is

$$\mathbf{s}' = c\mathbf{s}^F\mathbf{F}\mathbf{A} \circ [\mathbf{Q^1}, ..., \mathbf{Q^n}] \circ \mathbf{B}\mathbf{M} \cdot \mathbf{s}^M, \tag{14}$$

where $\mathbf{s}'$ is the phenotype share vector in the next generation for the target sex, assuming underlying additive effect of alleles. In our application, the target sex is males, but both sexes have the same share vector in many applications, as noted below.

What does this general expression look like for our lizard system? With six genotypes and Mendelian transmission, the following hex-matrix shows the probability that each genotype of progeny will be produced by a given pair of parental genotypes:

[1] Payoffs in finite two player games are typically given by bi-matrices. When only two of $K > 2$ players are strategically active, the payoffs can be specified nicely by K-matrices. Payoffs in general finite games are specified by multi-tensors. To our knowledge, Friedman and Sinervo (2016) is the first published example of using multi-matrices to specify payoffs arising from multi-locus bi-parental dynamics.

	Males:					
Fem:	oo	bo	yo	bb	by	yy
oo	[1,0,0,0,0,0]	$[\frac{1}{2},\frac{1}{2},0,0,0,0]$	$[\frac{1}{2},0,\frac{1}{2},0,0,0]$	[0,1,0,0,0,0]	$[0,\frac{1}{2},\frac{1}{2},0,0,0]$	[0,0,1,0,0,0]
bo	$[\frac{1}{2},\frac{1}{2},0,0,0,0]$	$[\frac{1}{4},\frac{1}{2},0,\frac{1}{4},0,0]$	$[\frac{1}{4},\frac{1}{4},\frac{1}{4},0,\frac{1}{4},0]$	$[0,\frac{1}{2},0,\frac{1}{2},0,0]$	$[0,\frac{1}{4},\frac{1}{4},\frac{1}{4},\frac{1}{4},0]$	$[0,0,\frac{1}{2},0,\frac{1}{2},0]$
yo	$[\frac{1}{2},0,\frac{1}{2},0,0,0]$	$[\frac{1}{4},\frac{1}{4},\frac{1}{4},0,\frac{1}{4},0]$	$[\frac{1}{4},0,\frac{1}{2},0,0,\frac{1}{4}]$	$[0,\frac{1}{2},0,\frac{1}{2},0,0]$	$[0,\frac{1}{4},\frac{1}{4},0,\frac{1}{4},\frac{1}{4}]$	$[0,0,\frac{1}{2},0,0,\frac{1}{2}]$
bb	[0,1,0,0,0,0]	$[0,\frac{1}{2},0,\frac{1}{2},0,0]$	$[0,\frac{1}{2},0,\frac{1}{2},0,0]$	[0,0,0,1,0,0]	$[0,0,0,\frac{1}{2},\frac{1}{2},0]$	[0,0,0,0,1,0]
by	$[0,\frac{1}{2},\frac{1}{2},0,0,0]$	$[0,\frac{1}{4},\frac{1}{4},\frac{1}{4},\frac{1}{4},0]$	$[0,\frac{1}{4},\frac{1}{4},0,\frac{1}{4},\frac{1}{4}]$	$[0,0,0,\frac{1}{2},\frac{1}{2},0]$	$[0,0,0,\frac{1}{4},\frac{1}{2},\frac{1}{4}]$	$[0,0,0,0,\frac{1}{2},\frac{1}{2}]$
yy	[0,0,1,0,0,0]	$[0,0,\frac{1}{2},0,\frac{1}{2},0]$	$[0,0,\frac{1}{2},0,0,\frac{1}{2}]$	[0,0,0,0,1,0]	$[0,0,0,0,\frac{1}{2},\frac{1}{2}]$	[0,0,0,0,0,1]

Table M: Genotype hex-matrix for Mendelian transmission

For example, the second diagonal entry $[\frac{1}{4},\frac{1}{2},0,\frac{1}{4},0,0]$ indicates offspring shares of a bo genotype female and the same genotype male: $\frac{1}{2}$ are also bo while $\frac{1}{4}$ are oo and $\frac{1}{4}$ are bb.

Since the observed female genotype data from one generation to the next will capture any impact of the female game on females, we can simplify some aspects of the structural model for male payoffs, which is our focus here. Thus we defer estimating a female game[2], which would more than double the number of parameters to estimate. By setting $\mathbf{F} = \mathbf{I}_6$, we capture the impacts of the female genotypes on male payoffs via their observed matings with males, and the male payoff matrix captures any strategic impacts on male progeny.

Our data do not permit identification of the mating preferences $\mathbf{A}, \mathbf{B}$ separately from the survival fitnesses $\mathbf{F}, \mathbf{M}$, so we write $\mathbf{A} = \mathbf{B} = \mathbf{1}_{n\times n}$ with the understanding that the empirical male survival matrix $\mathbf{M}$ also incorporates the impact of mating preferences as well. Male survival includes survival during egg development and from hatching to maturity (Lancaster *et al.* 2014, Sinervo *et al.* 1992, Sinervo *et al.* 2000). The female mating

[2]In particular, the density dependence in the phenotype fitness matrix is a proxy for female strategies. Female strategy shares are correlated over time with female population density (Sinervo et al. 2000), driven largely by a 2-year oscillation in the orange allele share. Furthermore, the female offspring size game that drives differences in the survival of female progeny, has a negligible impact on male progeny survival as experimentally demonstrated by Sinervo *et al.* (1992). These simplifications allow us to use density of adult males as the key parameter (from the female game) that impacts their payoffs because male territoriality (and their territorial strategies) is influenced by the overall male density in any given population (Scoular *et al.* 2011). Regardless, many of aspects of the female games influence on males per se will be collected in the male payoffs, as outlined in the text.

As noted in the text, we decompose overall fitness matrix via Hadamard products for temperature and density impacts on male payoffs: $\Omega = W \circ \exp[\beta^{\nu} \cdot \nu(t)] \circ \exp[\beta^{\tau} \cdot \tau(t)]$.

preferences subsume both direct female choice (Bleay & Sinervo 2007) and cryptic female choice (Calsbeek & Sinervo 2004), both of which operate in the side-blotched lizard system.

Assuming that survival (and mating preferences) of phenotypes are additively determined by underlying alleles, we write 6×6 genotype matrix $\mathbf{M}$ in terms of entries from the 3×3 phenotype fitness matrix expressing male versus male game payoffs $\mathbf{\Omega} = ((\omega_{ij}))$ as in Table N.

		Common:					
		O	OB	YO	B	BY	Y
Rare:		oo	bo	yo	bb	by	yy
O	oo	$\omega_{O,O}$	$\omega_{O,OB}$	$\omega_{O,YO}$	$\omega_{O,B}$	$\omega_{O,BY}$	$\omega_{O,Y}$
OB	bo	$\omega_{OB,O}$	$\omega_{OB,BO}$	$\omega_{OB,YO}$	$\omega_{OB,B}$	$\omega_{OB,BY}$	$\omega_{OB,Y}$
YO	yo	$\omega_{YO,O}$	$\omega_{YO,OB}$	$\omega_{YO,YO}$	$\omega_{YO,B}$	$\omega_{YO,BY}$	$\omega_{YO,Y}$
B	bb	$\omega_{B,O}$	$\omega_{B,OB}$	$\omega_{B,YO}$	$\omega_{B,B}$	$\omega_{B,BY}$	$\omega_{B,Y}$
BY	by	$\omega_{BY,O}$	$\omega_{BY,OB}$	$\omega_{BY,YO}$	$\omega_{BY,B}$	$\omega_{BY,BY}$	$\omega_{BY,Y}$
Y	yy	$\omega_{Y,O}$	$\omega_{Y,OB}$	$\omega_{Y,YO}$	$\omega_{Y,B}$	$\omega_{Y,BY}$	$\omega_{Y,Y}$

Table N: Genotype fitness matrix of males $\mathbf{M}$ written in terms of phenotype fitnesses ω.

Under the key assumption of additivity, all of the genotype payoffs ω in Table N can be re-expressed purely in terms of the homozygote payoffs (O,B,Y). Thus we have fitness $\frac{\omega_{II}+\omega_{IJ}}{2}$ in the case of rare homozygote I competing against common heterozygote IJ, or $\frac{\omega_{II}+\omega_{JI}}{2}$ in the case of a rare heterozygote type IJ competing against common homozygote type I. By additivity, the fitness of rare heterozygote type IJ competing against common heterozygote KL is $\frac{\omega_{IK}+\omega_{IL}+\omega_{JK}+\omega_{JL}}{4}$. See Sinervo (2001) for the original derivation of an equivalent expression.

The specification in Table O uses the most up to date information on female mating preferences for throat color and anti-predator dorsal patterns (Lancaster *et al.* 2009) and male endocrinology and behavior that suggests *bo* males are intermediate in phenotype between B and O phenotypes, being able to more aggressively defend territories than blue males (Calsbeek & Sinervo 2002), while *by* males are intermediate in phenotype between B and Y phenotypes, being able to cooperate (Sinervo *et al.* 2006) and sneak (Sinervo *et al.* 2000, Mills *et al.* 2008), depending on context, and that *yo* males are intermediate in phenotype between O and Y, being either territorial or sneaky, depending on social context

(Mills *et al.* 2008, Sinervo personal observations). The reader should note that these three heterozygous classes and the system of additivity will contribute to a rapid rise in frequency of the three strategies, even when homozygous classes are very low in frequency. Moreover, this structural model with sexual reproduction is analogous to the simple replicator model presented in the text.

		Common:					
		O	OB	YO	B	BY	Y
Rare:		oo	bo	yo	bb	by	yy
O	oo	ω_{OO}	$\frac{\omega_{OO}+\omega_{OB}}{2}$	$\frac{\omega_{OY}+\omega_{OO}}{2}$	ω_{OB}	$\frac{\omega_{OB}+\omega_{OY}}{2}$	ω_{OY}
OB	bo	$\frac{\omega_{OO}+\omega_{BO}}{2}$	$\frac{\omega_{OO}+\omega_{OB}+\omega_{BO}+\omega_{BB}}{4}$	$\frac{\omega_{OY}+\omega_{OO}+\omega_{BY}+\omega_{BO}}{4}$	$\frac{\omega_{OB}+\omega_{BB}}{2}$	$\frac{\omega_{OB}+\omega_{OY}+\omega_{BB}+\omega_{BY}}{4}$	$\frac{\omega_{OY}+\omega_{BY}}{2}$
YO	yo	$\frac{\omega_{YO}+\omega_{OO}}{2}$	$\frac{\omega_{YO}+\omega_{YB}+\omega_{OO}+\omega_{OB}}{4}$	$\frac{\omega_{YY}+\omega_{YO}+\omega_{OY}+\omega_{OO}}{4}$	$\frac{\omega_{YB}+\omega_{OB}}{2}$	$\frac{\omega_{YB}+\omega_{YY}+\omega_{OB}+\omega_{OY}}{4}$	$\frac{\omega_{YY}+\omega_{OY}}{2}$
B	bb	ω_{BO}	$\frac{\omega_{BO}+\omega_{BB}}{2}$	$\frac{\omega_{BO}+\omega_{BY}}{2}$	ω_{BB}	$\frac{\omega_{BB}+\omega_{BY}}{2}$	ω_{BY}
BY	by	$\frac{\omega_{BO}+\omega_{YO}}{2}$	$\frac{\omega_{BO}+\omega_{BB}+\omega_{YO}+\omega_{YB}}{4}$	$\frac{\omega_{BY}+\omega_{BO}+\omega_{YY}+\omega_{YO}}{4}$	$\frac{\omega_{BB}+\omega_{YB}}{2}$	$\frac{\omega_{BB}+\omega_{BY}+\omega_{YB}+\omega_{YY}}{4}$	$\frac{\omega_{BY}+\omega_{YY}}{2}$
Y	yy	ω_{YO}	$\frac{\omega_{YO}+\omega_{YB}}{2}$	$\frac{\omega_{YO}+\omega_{YY}}{2}$	ω_{YB}	$\frac{\omega_{YB}+\omega_{YY}}{2}$	ω_{YY}

Table O: Genotype fitness matrix $\mathbf{M}$ of males written in terms of the additive effect of alleles on phenotype fitnesses ω.

Thus, for lizards, the general equation now reads

$$\mathbf{s}' = c\mathbf{s}^F[\mathbf{Q}^{\mathbf{oo}}, ..., \mathbf{Q}^{\mathbf{yy}}]\mathbf{M} \cdot \mathbf{s}^M. \tag{15}$$

For example, the second component of $\mathbf{s}'$, for genotype bo, is given by the quadratic form $c\mathbf{s}^F\mathbf{Q}^{bo}\mathbf{M} \cdot \mathbf{s}^M$, where we see from Table M that $\mathbf{Q}^{bo} = \begin{pmatrix} 0 & \frac{1}{2} & 0 & 1 & \frac{1}{2} & 0 \\ \frac{1}{2} & \frac{1}{2} & \frac{1}{4} & \frac{1}{2} & \frac{1}{4} & 0 \\ 0 & \frac{1}{4} & 0 & \frac{1}{2} & \frac{1}{4} & 0 \\ 1 & \frac{1}{2} & \frac{1}{2} & 0 & 0 & 0 \\ \frac{1}{2} & \frac{1}{4} & \frac{1}{4} & 0 & 0 & 0 \\ 0 & 0 & 0 & 0 & 0 & 0 \end{pmatrix}$.

If $\mathbf{s}^F = \mathbf{s}^M = \mathbf{s}$, i.e., progeny of all genotypes have the usual 1:1 sex ratio, then we have $s'_2 = c\sum_{i,j=1}^{6} s_i s_j h_{ij}^{bo}$, where $\mathbf{H}^{bo} = \mathbf{Q}^{bo}\mathbf{M}$. One computes the other five components of s' similarly, using the corresponding quadratic forms $\mathbf{Q}^d\mathbf{M} = \mathbf{H}^d$ for $d = oo, ..., yy$. Of course, the normalization constant is chosen so that the six components sum to 1.0, i.e., c^{-1} is the sum of the unnormalized components.

Thus, given non-negative fitness values $\omega_{ij}, i, j \in \{O, B, Y\}$, and the current genotype vector, one can use equation (15) to predict the next phenotype vector. One fits the ω_{ij}s to

the data as described earlier, by finding values (plus a value of η, the Dirichlet parameter for precision or effective sample size) that maximize the likelihood of the observed data. Otherwise put, one replaces the Z expression obtained from deterministic replicator dynamics on the (phenotype) 3-simplex by an alternative Z expression obtained from (15) on the (genotype) 6-simplex.

Estimated parameters for diploid model. We estimated the diploid sexual model using data in Table 1 in two runs using different subsets of the data. Table P reports fitted parameters to the diploid model using male genotype data as inputs for both the male and female genotypes. This enables direct comparison to the featured replicator model, which only uses information from male genotypes. Table Q reports runs using both observed male and female genotypes as inputs. This allows us to assess the additional contribution of female genotypes per se to the payoffs of the male game. Using male only genotypes as a test is justified on the basis of the significant correlation observed between the sexes for each genotype (oo: $P < 0.05$, $t = 2.08$; bo: $P < 0.0001$, $t = 7.11$; yo: $P < 0.0001$, $t = 4.62$; bb: $P < 0.0001$, $t = 4.92$; by: $P < 0.0001$, $t = 10.08$; bo: $P < 0.0001$, $t = 7.11$; yy: $P < 0.0001$, $t = 5.23$).

Whereas the model with male only genotype data converges on estimates roughly similar payoffs to the replicator, when both the density and hours of restriction covariates are included, the model with female and male genotype converges on similar payoff estimates only when density is included as a covariate (i.e., covariate for hours of restriction excluded). In this case where density is the only covariate, the payoffs are similar to both the replicator and diploid sexual model with male only genotype data, with the additional feature of blue cooperation being recovered in the payoffs (e.g., b vs b has high fitness). In the case of the diploid sexual model run using both male and female genotype data, and including density and hours restriction covariates, the model appears to recover only high values for the payoffs of cooperation to the exclusion of all other terms of the payoff. The estimates are pretty fragile, can be quite different with different initializations of the optimization routine, and the likelihood function seems not well behaved. This behavior of the ML model when many covariates are input is a strong argument for better data inputs. For example, inspection of the data shows a rapid rise in b alleles (in both males and females) in only a limited number of years. Thus, the fragility of the estimates when

including hours of restriction covariates, is likely directly related to the limited number of years (e.g., cases of hours of restriction) in which we have observed a striking positive frequency dependent rise in the shares of the b allele (MW: 1992-1993, 1998-1999; MW, OW, YW, BW: 2002-2003, MW, OW, YW: 2008-2009). Thus, this suggests that additional data on numbers of populations and years of climate data will be required to further resolve the payoffs of the female genotypes per se, along with an explicit payoff matrix for the female games influence on genotypes in the next generation. In addition, the larger values for SEs for the estimates (relative to payoffs and covariates) compared to the replicator SEs for payoffs and covariates, suggests that this more complex diploid model will require additional data to resolve these more nuanced between sex and within morph (cooperation) interactions. Nevertheless, the similarity of the diploid sexual model to the replicator model, when used with the male only genotypes, indicates our primary results on the male genotypes per se are robust to the choice of the underlying structural equation model employed for the male game.

(a) Estimated W

0.0000 (0.2006)	0.3704 (0.1292)	0.0000 (0.1595)
0.1157 (0.2742)	0.0000 (0.2436)	0.0001 (0.2278)
0.5136 (0.1464)	0.0000 (0.1456)	0.0000 (0.1858)

(b) Estimated β_ν

4.0892 (51.37)	-10.6680 (41.45)	-59.3954 (44.85)
14.8576 (47.77)	4.3651 (53.06)	4.8786 (46.47)
11.9609 (46.77)	24.8565 (46.23)	5.0555 (64.52)

(c) Estimated β_τ

55.4512 (72.86)	37.6807 (59.97)	-47.9446 (69.08)
-10.0251 (73.88)	-18.7374 (91.67)	52.4157 (64.87)
-15.1855 (75.21)	-107.5945 (54.51)	53.9396 (75.41)

Table P: Estimates of the $(W, \beta_\nu, \beta_\tau)$ for the diploid sexual model run with male genotype data used for both female and male inputs. Estimated effective sample size is $\hat{\eta} = 14.2 \pm 1.1$.

(a) Estimated W

0.00 (0.00)	0.001 (0.00)	0.64 (0.002)
0.00 (0.00)	0.00 (0.00)	0.35 (0.002)
0.00 (0.00)	0.00 (0.00)	0.01 (0.00)

(b) Estimated β_ν

13.39 (4.24)	2.78 (4.24)	-36.81 (4.24)
-1.26 (33.91)	12.96 (4.24)	-14.09 (4.24)
13.07 (4.24)	13.11 (4.24)	-3.14 (4.24)

(c) Estimated W

0.23 (0.07)	0.00 (0.12)	0.00 (0.16)
0.28 (0.14)	0.001 (0.00)	0.004 (0.12)
0.48 (0.00)	0.00 (0.11)	0.00 (0.05)

(d) Estimated β_ν

5.43 (91.33)	24.19 (100.38)	-10.23 (133.48)
-9.13 (149.91)	17.62 (43.96)	21.64 (140.08)
-18.45 (43.94)	-49.96 (48.28)	18.88 (55.60)

(e) Estimated β_τ

-19.3919 (58.74)	-56.7714 (56.67)	-42.7855 (78.42)
18.0420 (86.91)	71.2613 (18.92)	44.5500 (79.49)
-3.2700 (18.88)	-9.8699 (22.96)	-1.7645 (32.78)

Table Q: Estimates of matrices W (Panel a) and β_ν (Panel b) for the diploid sexual model run with male and female genotype data, ignoring hours of restriction ($\beta_\tau = 0$). Remaining panels show estimates for that model including hours of restriction; estimated effective sample size is $\hat{\eta} = 11.85 \pm 0.10$.

Phylogeographic variation of the OBY locus and climate forced fixation. We used the published data on the OBY locus (Corl *et al.* 2010) and a published ecophysiological model of climate forcing effects (Sinervo *et al.* 2010). To apply our estimated replicator model for climate sensitive payoffs due to hours of restriction, we first estimated the quadratic relationship between monthly h_r and monthly $T_{max} - T_b$

($T_b = 37.4$, Paranjpe *et al.* 2013) at Los Banos during the months of June, July and August and spanning the years 1989 to 2011 (T_{max} from weather stations described in the text). The fitted equation is

$h_r = 1.6753291 + (T_{max} - T_b)0.2515211 + (T_{max} - T_b + 4.93991)(T_{max} - T_b + 4.93991)0.040644$ for $T_{max} - T_b > 8^o$C, and $h_r = 0$ for $T_{max} - T_b \leq 8^o$C.

Latitude	Longitude	Elevation	Species	Tb	Source
36.99	-121.05	271	Uta stansburiana	36	Baastians and Sinervo
31.85	-103.09	872	Uta stansburiana	37	Ferguson 1971
31.15	-108.73	1377	Uta stansburiana	36	Ferguson 1971
40	-119.44	1618	Uta stansburiana	38	Ferguson 1971
40.84	-112.03	1283	Uta stansburiana	34	Ferguson 1971
34.36	-117.2	1126	Uta stansburiana	36	Ferguson 1971
32.69	-116.97	241	Uta stansburiana	37	Ferguson 1971
28.7	-112.57	162	Uta stansburiana	37	Ferguson 1971
42.6	-115.7	1160	Uta stansburiana	35.56	Parker and Pianka 1975
40.6	-112.499	1369	Uta stansburiana	35.46	Parker and Pianka 1975
40.2	-118.45	1221	Uta stansburiana	34.78	Parker and Pianka 1975
38.8	-117.97	1395	Uta stansburiana	35.47	Parker and Pianka 1975
37.09	-116.95	1260	Uta stansburiana	35	Parker and Pianka 1975
36.3	-115.786	1771	Uta stansburiana	34.79	Parker and Pianka 1975
35.3	-114.86	790	Uta stansburiana	34.59	Parker and Pianka 1975
35.1	-118.164	1145	Uta stansburiana	35.69	Parker and Pianka 1975
35.1	-118.164	1145	Uta stansburiana	34.05	Parker and Pianka 1975
34.111	-116.57	1285	Uta stansburiana	36.2	Parker and Pianka 1975
34.111	-116.57	1285	Uta stansburiana	36.4	Parker and Pianka 1975
33.69	-113.01	380	Uta stansburiana	35.95	Parker and Pianka 1975
33.69	-113.01	380	Uta stansburiana	35.84	Parker and Pianka 1975
32.95	-111.98	385	Uta stansburiana	35.84	Parker and Pianka 1975
32.95	-111.98	385	Uta stansburiana	35.19	Parker and Pianka 1975
42.6	-115.7	1160	Uta stansburiana	35.21	Parker and Pianka 1975
35.3	-114.86	790	Uta stansburiana	35.55	Parker and Pianka 1975
24.46	-110.36	20	Uta stansburiana	36.2	Soule 1963

Table R: Geographic variation in body temperature. Citations can be found in Sinervo *et al.* (2010)

Next, to estimate T_b by latitude (Lat), we used the fitted equation $T_b = -0.109436 * Lat + 39.489642$ derived from data reported in (Sinervo *et al.* 2010) and reproduced in Table R below. (N.B., there was one outlier in the Ferguson [1971] cited in Sinervo *et al.* 2010 data set and we ran the model excluding and including the outlier for

T_b. Results were similar and here we report the results excluding the outlier.) These two equations allowed us to compute h_r across the species range using the monthly T_{max} during the hatchling emergence period. We estimated the months of hatchling emergence directly from observations on months of reproduction for the 1st clutch at 27 sites located across the species range (Mapimi, Mexico; Crane County, TX; Corn Springs, CA; Pisgah Crater, CA; Granite Mountains, CA; Death Valley, CA; Darwin Falls, CA; Table Mountain, CA; Largo Vista, Pearblossom, CA; Day Light, NV; Lovelock, NV; Sedgwick, CA; Anacapa Island, CA; Santa Cruz Island, CA; Pozo Road, CA; Naciemento Road, CA; Zayante Road, CA; Alamo Mountain, CA; Pinnacles National Park, CA; Los Banos, CA; Del Puerto Canyon Road, CA; Corral Hollow Road, CA; Mercury Springs, OR; Burns, OR; Deschutes River, Terrebonne, OR; Vantage, WA).

We used this information to estimate the reproductive period of the 1st clutch at nearby sites, at a comparable latitude, longitude, and elevation. The hatchling period spanning 3 months was estimated to start 2 months after the start of the reproductive period, since it takes 2 months to yolk a clutch and for eggs to incubate. We computed average h_r for each site using the T_{max} from worldClim.org for conditions from 1950 to 2000, the T_b from the regression relationship across the *Uta* range and we standardized h_r to the h_r computed for the Los Banos site to obtain τ in the climate driven payoff matrix for each site across the species range. Values for fitted T_b and fitted h_r, observed OBY alleles, and month for the start of the reproductive period are reported in Table S.

Site	Lat	Lon	Tb	h_r	o	b	y	Month
Los Banos	37.00	-121.05	35.44	1.10	0.12	0.49	0.39	4
Corral Hollow	37.64	-121.49	35.37	1.09	0.03	0.50	0.47	4
Pinnacles	36.49	-121.17	35.50	0.19	0.40	0.43	0.17	4
Big Creek	36.09	-121.60	35.54	0.00	0.50	0.50	0.00	4
Nacimiento Rd	35.99	-121.41	35.55	0.00	0.50	0.50	0.00	4
Sedgwick	34.74	-120.03	35.69	0.00	0.15	0.67	0.17	3
Santa Cruz Island	34.04	-119.57	35.76	0.00	0.41	0.46	0.13	4
Anacapa Island	34.01	-119.37	35.77	0.00	0.02	0.98	0.00	4
Stunt Ranch	34.11	-118.65	35.76	0.00	0.29	0.41	0.30	3
Power	34.82	-116.34	35.68	0.51	0.36	0.54	0.11	2
Pisgah Lava Flow	34.76	-116.37	35.69	0.51	0.28	0.72	0.00	2
Granite Mountains	34.79	-115.64	35.68	1.52	0.17	0.62	0.21	3
Corn Springs	33.62	-115.32	35.81	0.84	0.25	0.58	0.17	2
Mountain Springs	32.67	-116.10	35.91	0.23	0.43	0.55	0.02	2
Kofa NWR	33.25	-114.22	35.85	0.96	0.17	0.68	0.15	2
McDowell Mountains	33.67	-111.87	35.81	1.42	0.29	0.62	0.10	2
White Sands NM	32.79	-106.22	35.90	0.31	0.50	0.44	0.06	2
Guadalupe Mountains NP	31.92	-105.00	36.00	0.54	0.37	0.50	0.13	4
Bitter Lake NWR	33.58	-104.39	35.82	1.25	0.43	0.57	0.00	3
Carmen Island	26.02	-111.16	36.64	1.31	0.20	0.80	0.00	3
Danzante Island	25.78	-111.26	36.67	0.99	0.07	0.57	0.36	3
Monserrat Island	25.68	-111.02	36.68	1.68	0.44	0.44	0.13	3
Santa Catalina Island	25.61	-110.79	36.69	1.43	0.28	0.54	0.19	3
San Jose Island	24.90	-110.58	36.77	0.92	0.32	0.55	0.13	3
San Francisco Island	24.83	-110.58	36.77	1.11	0.30	0.43	0.28	3
Petrified Forest National Park	34.96	-109.79	35.66	0.31	0.50	0.50	0.00	3
Wupatki National Monument	35.53	-111.32	35.60	0.99	0.50	0.50	0.00	3
Zion National Park	37.17	-113.03	35.42	0.21	0.50	0.50	0.00	3
Colorado NM	39.10	-108.71	35.21	0.42	0.50	0.50	0.00	4
Dinosaur NM	40.42	-109.19	35.07	0.48	0.50	0.50	0.00	4
Mercury	36.59	-116.00	35.49	0.57	0.33	0.45	0.23	3
Lytle Ranch	37.08	-113.93	35.43	0.65	0.30	0.57	0.13	3
Delta	39.70	-113.10	35.15	0.42	1.00	0.00	0.00	4
Grantsville	40.60	-112.54	35.05	0.05	1.00	0.00	0.00	4
Darwin Falls	36.32	-117.52	35.51	0.89	0.20	0.57	0.23	4
Daylight Pass	36.80	-116.92	35.46	1.34	0.17	0.62	0.21	3
Lovelock	40.27	-118.54	35.08	0.46	1.00	0.00	0.00	4
Warner Mountains	41.35	-120.13	34.96	0.15	1.00	0.00	0.00	4
Burns	43.44	-118.93	34.74	0.06	1.00	0.00	0.00	4
Horseridge	43.96	-121.05	34.68	0.00	1.00	0.00	0.00	4
Vantage	46.90	-119.95	34.36	0.50	1.00	0.00	0.00	4

Table S: Geographic variation in computed body temperature, and computed hours of restriction during the hatchling period, observed OBY allele frequency from (Corl *et al.* 2010) and month of the 1st clutch.

1 Bibliography

1. Alonzo, S.H. & Sinervo, B. (2001) Mate choice games, context-dependent good genes, and genetic cycles in the side-blotched lizard *Uta stansburiana. Behav. Ecol. Sociobiol.* 49:176-186.

2. Bleay, C. & Sinervo, B (2007) Discrete genetic variation in mate choice and a condition dependent preference function in the side blotched lizard: Implications for the formation and maintenance of co-adapted gene complexes. *Behavioral Ecology* 18: 304-310.

3. Calsbeek, R. & Sinervo, B. (2002) The ontogeny of territoriality during maturation. *Oecologia* 132: 468-477.

4. Calsbeek, R. & Sinervo, B. (2004) Within-clutch variation in offspring sex determined by differences in sire body size: cryptic mate choice in the wild. *Journal of Evolutionary Biology* 17: 464-470.

5. Corl, A., Davis, A.R., Kuchta, S.R., & Sinervo, B. (2010) Selective loss of polymorphic mating types is associated with rapid phenotypic evolution during morphic speciation. *Proc Natl Acad Sci USA* 107: 4294-4259.

6. Friedman, D. & Sinervo, B. (2016) *Evolutionary Games in Natural, Social and Virtual Worlds.* Oxford University Press, New York, NY, USA.

7. Davidson, R. & MacKinnon, J.R. (2004) *Econometric Theory and Methods.* Oxford, UK: Oxford University Press.

8. Kotz, S., Balakrishnan, N., & Johnson, N. L. (2000) *Continuous Multivariate Distributions.* Volume 1: Models and Applications. New York: Wiley. (Chapter 49).

9. Lancaster, L., Hipsley, C., & Sinervo, B. (2009) Female choice for optimal combinations of multiple male display traits increases offspring survival. *Behav. Ecol.* 20: 993-999.

10. Lancaster, L., McAdam, A. G., Hipsley, C., & B. Sinervo. 2014. Frequency-dependent and correlational selection pressures have conflicting consequences for assortative mating in a color-polymorphic lizard, Uta stansburiana. *American Naturalist* 184:188-197.

11. Mills, S., Hazard, L., Lancaster, L., Mappes, T., Miles, D. B., Oksanen, T. & Sinervo,

B. (2008) Gonadotropin hormone modulation of testosterone, immune function, performance, and behavioral trade-offs among male morphs of the lizard, *Uta stansburiana. Am. Nat.*, 171: 339-357.

12. Paranjpe, D.A., Bastiaans, E., Patten, A., Cooper, R.D., & Sinervo, B. (2013) Evidence of maternal effects on temperature preference in side-blotched lizards: implications for evolutionary response to climate change. *Ecol. Evol.* doi: 10.1002/ece3.614.

13. Scoular, K. M., Caffry, W. C., Tillman, J. L., Finan, E. S., Scwartz, S. K., Sinervo, B. & Zani, P. (2011) Multiyear home-range ecology of common side-blotched lizards in Eastern Oregon with additional analysis of geographic variation in home-range size. *Herpetological Monographs* 25: 52-75.

14. Sinervo, B. (2001) Runaway social games, genetic cycles driven by alternative male and female strategies, and the origin of morphs. *Genetica* 112:417-434.

15. Sinervo, B., Miles, D.B., Frankino, W.A. Klukowski, M., & DeNardo, D.F. (2000) Testosterone, endurance, and Darwinian fitness: natural and sexual selection on the physiological bases of alternative male behaviors in side-blotched lizards. *Hormones and Behavior* 38: 222-233.

16. Sinervo, B., Bleay, C., & Adamopoulou, C. (2001) Social causes of correlational selection and the resolution of a heritable throat color polymorphism in a lizard. *Evolution* 240:2040-2052.

17. Sinervo, B., Chaine, A., Clobert, J., Calsbeek, R.G., McAdam, A., Hazard, L., *et al.* (2006) Color morphs and genetic cycles of greenbeard mutualism and transient altruism. *Proc. Natl. Acad. Sci. USA* 102:7372-7377.

18. Sinervo, B., Heulin, B., Surget-Groba, Y., Clobert, J., Corl, A., Chaine, A., *et al.* (2007) Models of density-dependent genic selection and a new Rock-Paper-Scissors social system. *Am. Nat.* 170: 663-680.

19. Sinervo, B, Mendez-de-la-Cruz, F.R. Miles, D.B., Heulin, B., Bastiaans, E., Villagran-Santa Cruz, M. *et al.* (2010) Erosion of lizard diversity by climate change and altered thermal niches. *Science* 324:894-899.

20. Sinervo, B., Svensson, E.I., & Comendant, T. (2000) Density cycles and an offspring

quantity and quality game driven by natural selection. *Nature* 406:985-988.

21. Sinervo, B., Doughty, P., Huey, R.B. & Zamudio, K. (1992) Allometric engineering: A causal analysis of natural selection on offspring size. *Science* 258: 1927-1930.
