# Supplementary material for: Evolutionary games, climate and the generation of diversity
Source: PLoS One. 2017 Aug 31;12(8):e0184052. doi: 10.1371/journal.pone.0184052 (PMC5578633; doi:10.1371/journal.pone.0184052)
Supplement: S1 Code — (ZIP) [file pone.0184052.s003.zip › comments.pdf]

Link to the matlab code folder:
https://www.dropbox.com/sh/d5baa6k9yw8gjmo/AACTPmWPX20RvgQGiQcYo0oma?oref=e

**DATA**
Files: newgenodata(121715).mat
Comment:
This Matlab data file contains five variables:

- FS: a 6-by-65 matrix of female shares
- ID: a 1-by-65 vector of world identifiers (1=MW,2=OW,3=BW,4=YW)
- MS: a 6-by-65 matrix of male shares
- hr: a 1-by-65 vector of hours of restrictions
- pd: a 1-by-65 vector of population densities

**ESTIMATION CALLING PROGRAMS**
Files: estimation_model1.m, estimation_model2.m, estimation_model3.m., estimation_model1_maleonly.m, estimation_model2_maleonly.m, estimation_model3_maleonly.m.,
Comment:
Each of these files calls the estimation of a model. Model 1 is the basic model, model 2 is the model augmented with population density and model 3 is the model augmented with population density and hours of restriction. The "_maleonly" versions use only data on male lizards. Each file uses the main data file and saves the estimation results in a new file. Estimation can take some time for the augmented models. See the comments in each Matlab file.

**STANDARD ERROR BOOSTRAP CALLING PROGRAMS**
Files: sebootstrap_model1.m, sebootstrap_model2.m, sebootstrap_model3.m, sebootstrap_model1male.m, sebootstrap_model2male.m, sebootstrap_model3male.m.
Comment:
Each of these files calls the computation of the standard errors of the estimates of a model. The "male" versions use only data on male lizards. Each file uses the main data file and the file containing the estimation results (so you need to run the estimation first). It then saves a new file containing the standard errors. Estimation can take some time for the augmented models. See the comments in each Matlab file.

**LIKELIHOOD FUNCTIONS**
Files: LF1.m, LF2.m, LF3.m.
Comment:
Each of these files defines a Matlab function that computes the log likelihood of a model given parameters and data. The main idea is the following. We assume that the period $t$ 6-by-1 genotype share vector $s_t$ is distributed as a Dirichlet random variable with parameters given by the 6-by-1 vector $\eta z_t$, where $z_t$ is the deterministic dynamics derived from the model. Given $\eta z_t$ we can compute the likelihood in the usual way (see equations 5,6,7).

**DETERMINISTIC DYNAMICS FUNCTION**

Files: detdyn.m

Comment:

This Matlab function computes the deterministic dynamics $z_t$ according to the main equation of the diploid model (eq. 20 of the SI). The computation is done in the following way: given matrices $Q$ and $M$ and share vectors $x$ and $y$, first for each $i = 1,\ldots,6$ compute: $a_i = xQ^i My$; then compute $z_{it} = \frac{a_{it}}{\sum_j a_{jt}}$.

So for example in our basic model, with $x = s^f_{t-1}$ and $y = s^m_{t-1}$, we have $z_{it} = \frac{s^f_{t-1} Q^i M s^m_{t-1}}{\sum_j s^f_{t-1} Q^j M s^m_{t-1}}$

**FUNCTION FOR CREATING THE MATRIX M**

Files: mapM.m

Comment:

This Matlab function creates the matrix $M$ from the payoff matrix $W$ or augmented payoff matrix $\Omega$ (which in turn depends on the actual payoff matrix $W$ but also on time-varying variables), according to a given mapping, that can be either dominance-based or additive. This file includes both mappings: just (manually) comment out the one that is not relevant.

**FUNCTION FOR CREATING THE MATRIX Q**

Files: Qmatrix.m

Comment: When called (without any inputs) this function just creates $Q$.

**DIRICHLET SAMPLING FUNCTION**

Files: sample_dirichlet.m

Comment: This function draws a random Dirichlet distributed vector. There are two places where we use this:

1. simulations of dynamics: since we assume that the 6-by-1 share vector $s^m_t$ is distributed as a Dirichlet random variable with parameters given by the 6-by-1 vector $\eta z_t$, given $\eta z_t$ we can simulate the stochastic dynamics in the next period using this function. We use this simulation procedure for bootstrapping the standard errors.
2. when we need to draw a random payoff matrix to initialize the optimization of the likelihood function, we draw them using a Dirichlet distribution (so this is completely unrelated to our main distributional assumption and to point 1 above…).
